# The Somatostatin 2A Receptor Is Enriched in Migrating Neurons during Rat and Human Brain Development and Stimulates Migration and Axonal Outgrowth

**DOI:** 10.1371/journal.pone.0005509

**Published:** 2009-05-12

**Authors:** Virginia Le Verche, Angela M. Kaindl, Catherine Verney, Zsolt Csaba, Stéphane Peineau, Paul Olivier, Homa Adle-Biassette, Christophe Leterrier, Tania Vitalis, Julie Renaud, Bénédicte Dargent, Pierre Gressens, Pascal Dournaud

**Affiliations:** 1 Inserm, Unité Mixte de Recherche U676, Paris, France; 2 Université de Médecine Denis Diderot-Paris 7, Paris, France; 3 MRC centre for Synaptic Plasticity, Department of Anatomy, Bristol, United Kingdom; 4 Inserm, Unité Mixte de Recherche 641, Marseille, France; 5 Université de la Méditerranée, Faculté de Médecine Secteur-Nord, Institut Fédératif de Recherche 11, Marseille, France; 6 Ecole Supérieure de Physique et de Chimie Industrielles–CNRS 7537, Paris, France; 7 Inserm, Unité Mixte de Recherche S968, Institut de la Vision, Department of Development, Paris, France; 8 Université Pierre et Marie Curie-Paris 6, Institut de la Vision, Paris, France; Columbia University, United States of America

## Abstract

The neuropeptide somatostatin has been suggested to play an important role during neuronal development in addition to its established modulatory impact on neuroendocrine, motor and cognitive functions in adults. Although six somatostatin G protein-coupled receptors have been discovered, little is known about their distribution and function in the developing mammalian brain. In this study, we have first characterized the developmental expression of the somatostatin receptor sst2A, the subtype found most prominently in the adult rat and human nervous system. In the rat, the sst2A receptor expression appears as early as E12 and is restricted to post-mitotic neuronal populations leaving the ventricular zone. From E12 on, migrating neuronal populations immunopositive for the receptor were observed in numerous developing regions including the cerebral cortex, hippocampus and ganglionic eminences. Intense but transient immunoreactive signals were detected in the deep part of the external granular layer of the cerebellum, the rostral migratory stream and in tyrosine hydroxylase- and serotonin- positive neurons and axons. Activation of the sst2A receptor *in vitro* in rat cerebellar microexplants and primary hippocampal neurons revealed stimulatory effects on neuronal migration and axonal growth, respectively. In the human cortex, receptor immunoreactivity was located in the preplate at early development stages (8 gestational weeks) and was enriched to the outer part of the germinal zone at later stages. In the cerebellum, the deep part of the external granular layer was strongly immunoreactive at 19 gestational weeks, similar to the finding in rodents. In addition, migrating granule cells in the internal granular layer were also receptor-positive. Together, theses results strongly suggest that the somatostatin sst2A receptor participates in the development and maturation of specific neuronal populations during rat and human brain ontogenesis.

## Introduction

Only a restricted number of neuropeptides have been reported to play a role in the fetal and early post-natal brain, among the region-specific factors that control cell proliferation, migration and differentiation during brain development. This is in sharp contrast with the extensive literature reporting various and robust physiological functions of neuropeptides in the adult central nervous system. The relative lack of specific antibodies against neuropeptide receptors might account for such discrepancy. Precise information on the regional and cellular localization of receptors is indeed required to ascribe a potential neurodevelopmental role to a given neuropeptide.

The neuropeptide somatostatin (somatotropin release inhibiting factor, SRIF) [1] has a wide variety of biological roles [1]–[3]. In the adult brain, SRIF regulates neuroendocrine, motor and cognitive functions [4], [5]. Perturbation of somatostatinergic neurotransmission has been demonstrated in temporal lobe epilepsy [6]–[8], ischemia [9], [10] and Alzheimer's disease [11].

The five SRIF receptors (sst1–sst5) belong to the family of G protein-coupled receptors (GPCRs) and bind the native peptides SRIF-14, SRIF-28 and the more recently discovered neuropeptide cortistatin [12] with high affinity [1], [13]. While sst1, sst3, sst4 and sst5 genes each generate a single receptor protein, alternative splicing of the sst2 mRNA gives rise to two protein isoforms, sst2A and sst2B [14], [15]. In the adult mammalian brain, converging evidence suggests that the sst2A receptor exerts a predominant role in the transduction of SRIF actions [1], [4], [13].

In the developing brain, there is now evidence that like the pituitary adenylate cyclase-activating peptide (PACAP) [16] and neuropeptide Y (NPY) [17], [18], SRIF may also play an important role in neuronal development [19]. This could be mediated by the sst2 receptor type since sst2 receptor mRNA [20]–[22] and binding sites [23]–[28] are predominant in the developing rat and human brain. In addition, the sst2 receptor gene has recently been demonstrated to be in the top 40 genes (out of 20 000) up-regulated during neuronal development [29], suggesting a specific role for this receptor during this period.

Because the ontogenic distribution of the sst2A receptor at the protein level has not yet been determined, the aim of the present study was to localize this receptor during rat brain ante- and post-natal development (E10-P21). Sst2A receptor distribution was studied in parallel in the human prenatal cerebral cortex and cerebellum from gestational week 8 to birth. In an attempt to elucidate the functional role of the sst2A receptor during early neuronal development, the effect of sst2A receptor activation on neuronal migration and neurite patterning was also analyzed *in vitro* in cerebellar microexplants and primary hippocampal neurons, respectively.

## Results

### Rhombencephalon and cerebellum

**Figure 1:**
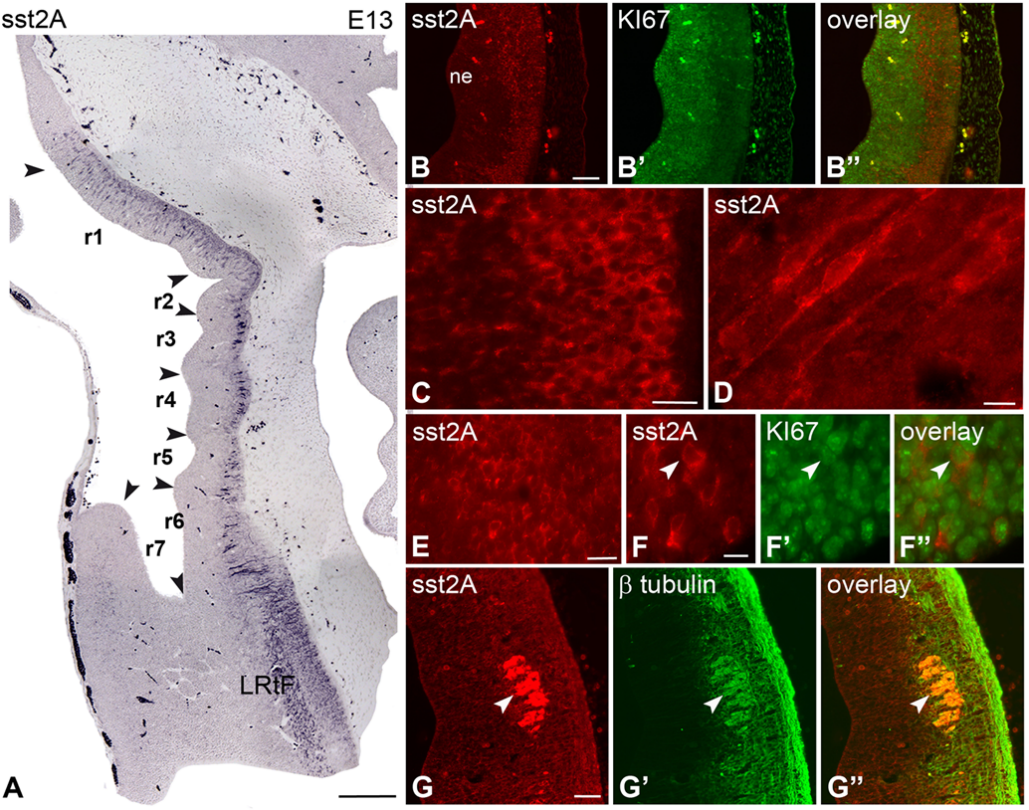
Regional and cellular localization of the sst2A receptor immunoreactivity in sagittal sections of the rat rhombencephalon at embryonic day 13 (E13). A) Densely packed sst2A receptor-immunoreactive cells are observed in the marginal zone contiguous to the ventricular zone of the rhombomeres (r1 to r6, arrowheads) and in the lateral reticular formation (LRtF). B–B″) Sst2A receptor-immunoreactive cells (red) are localized in the marginal zone (B) whereas proliferating cells identified by the proliferation marker Ki-67 (green) are concentrated in the ventricular zone (B′). The lack of overlap between the two signals (B″) indicates that sst2A receptor-expressing cells are predominantly post-mitotic. C) The majority of sst2A receptor-immunoreactive cells have small round perikarya and some exhibit immunolabeled processes that are oriented perpendicularly to the ventricular surface. D) A few sst2A receptor-immunoreactive cells are bipolar, displaying the morphological features of migrating neurons. E) In the LRtF, cell bodies are strongly sst2A receptor-immunoreactive. F–F″) An sst2A receptor-immunoreactive cell (red in F, F″) of the LRtF is found to be Ki-67-positive (green in F′, F″) (arrowheads). The low percentage of colocalization (F″) indicates that the majority of receptor-expressing cells are post-mitotic. G–G″) The post-mitotic feature of most sst2A receptor-immunoreactive cells (red in G, G″) of the rhombencephalon is further indicated by the colocalization (G″) with the post-mitotic neuronal marker β-tubulin (green in G′, G″) (arrowheads), as illustrated in the facial nucleus. Scale bars: A, 250 µm; B–B″, G–G″, 50 µm; C, E, 20 µm; F, 10 µm.

**Figure 2:**
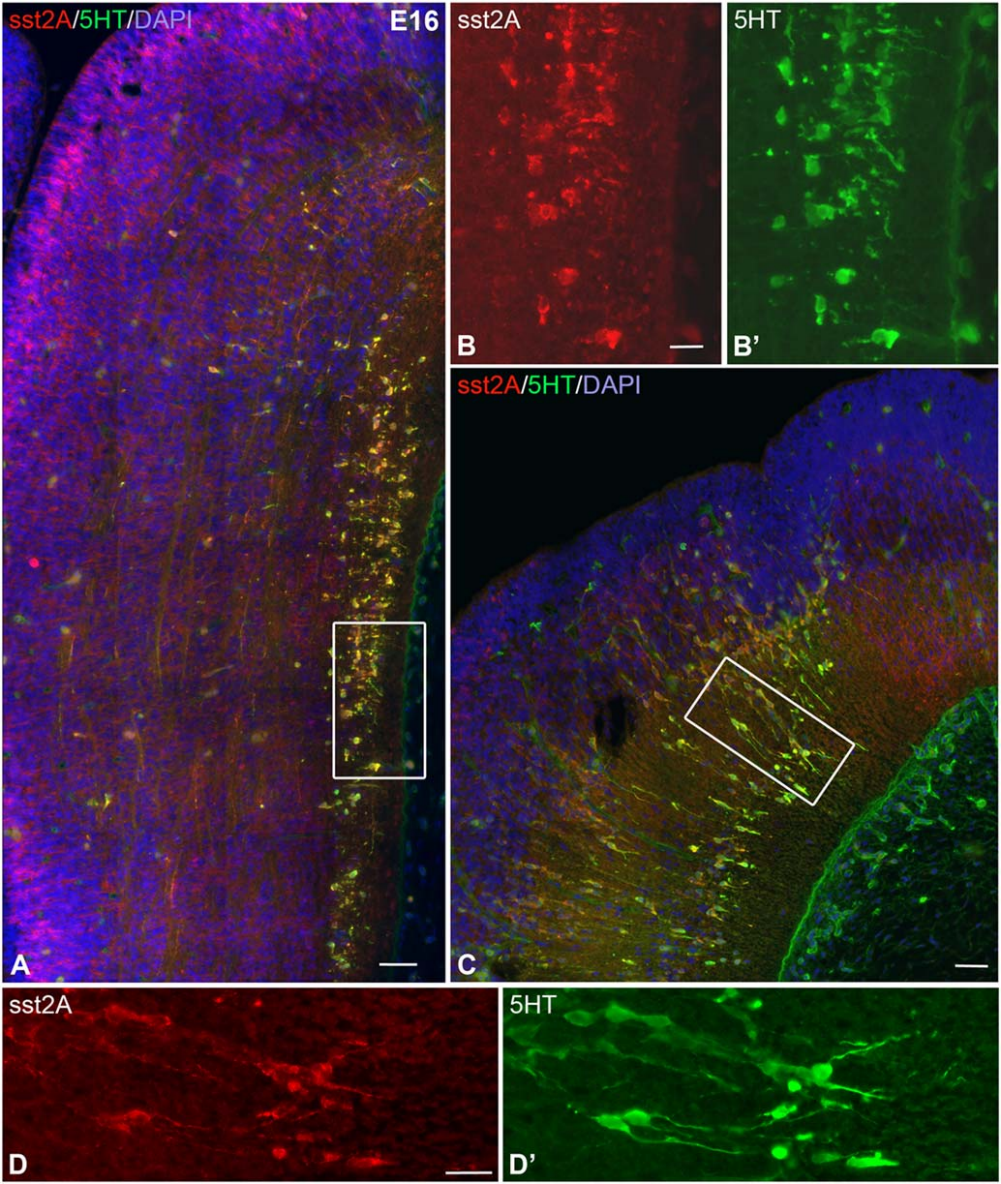
Expression of the sst2A receptor in serotoninergic neurons of the rat brainstem at E16. A) Triple-labeling with sst2A receptor (red), 5-HT (green) and DAPI (blue) in the ventral part of the brainstem illustrates that most serotoninergic neurons are sst2A receptor immunoreactive. B,B′ represent magnification of the boxed area in A. Note the extensive colocalization of sst2A receptor and 5-HT in both cell bodies and processes. C) The sst2A receptor is also expressed in serotoninergic migrating cells in the more dorsal part of the mesencephalon presumably corresponding to the dorsal raphe nucleus. D,D′ represents magnification of the boxed area in C and illustrates double-labeling in both the soma and processes of migrating neurons. Scale bars: A, C, 50 µm; B, D, 20 µm.

Cells expressing the sst2A receptor were first detected at E13 in the rhombomeres (Fig. 1). Round, small (∼7 µm) and densely packed immunoreactive cells were located along the outer limit of the ventricular zone, in the marginal zone, throughout rhombomeres r1 to r6. Immunoreactive processes were apparent in the ventricular zone, extending perpendicularly to the ventricle. Only rare colocalization with the proliferation marker Ki-67 was observed demonstrating that most of sst2A receptor-positive cells were not proliferating ones (Fig. 1B–B″, F–F″). Double-labeling with the β-tubulin marker confirmed that this population of sst2A receptor-expressing cells was mostly post-mitotic neurons. In addition to this cell population, the sst2A receptor was also expressed by round and small post-mitotic neurons extending caudally to the nascent reticular formation and spinal cord. At E13, intense sst2A receptor immunoreactivity was visualized in the roots of several cranial nerves including the facial (7n) (Fig. 1G–G″), trigeminal and cochlear nerves. This labeling was no longer observed after E15. From E13 to E16, the number of sst2A receptor expressing cells became higher in the lateral reticular formation. These cells also expressed the β-tubulin marker. Between E15 and E17, most of serotonin (5-HT) positive cells, forming longitudinal columns on both sides of the floor plate from the developing dorsal raphe nucleus toward the raphe magnus nucleus, were immunoreactive for the sst2A receptor (Fig. 2). Neuronal processes as well as longitudinal and transversal fibers immunoreactive for 5-HT were positive for sst2A receptor immunoreactivity (Fig. 2D–D′). Rhombencephalic serotoninergic neurons also expressed the receptor. From E17 onwards, sst2A receptor immunoreactivity decreased gradually in the developing brainstem. Only very weak receptor immunoreactivity was apparent at E21 throughout brainstem nuclei.

**Figure 3:**
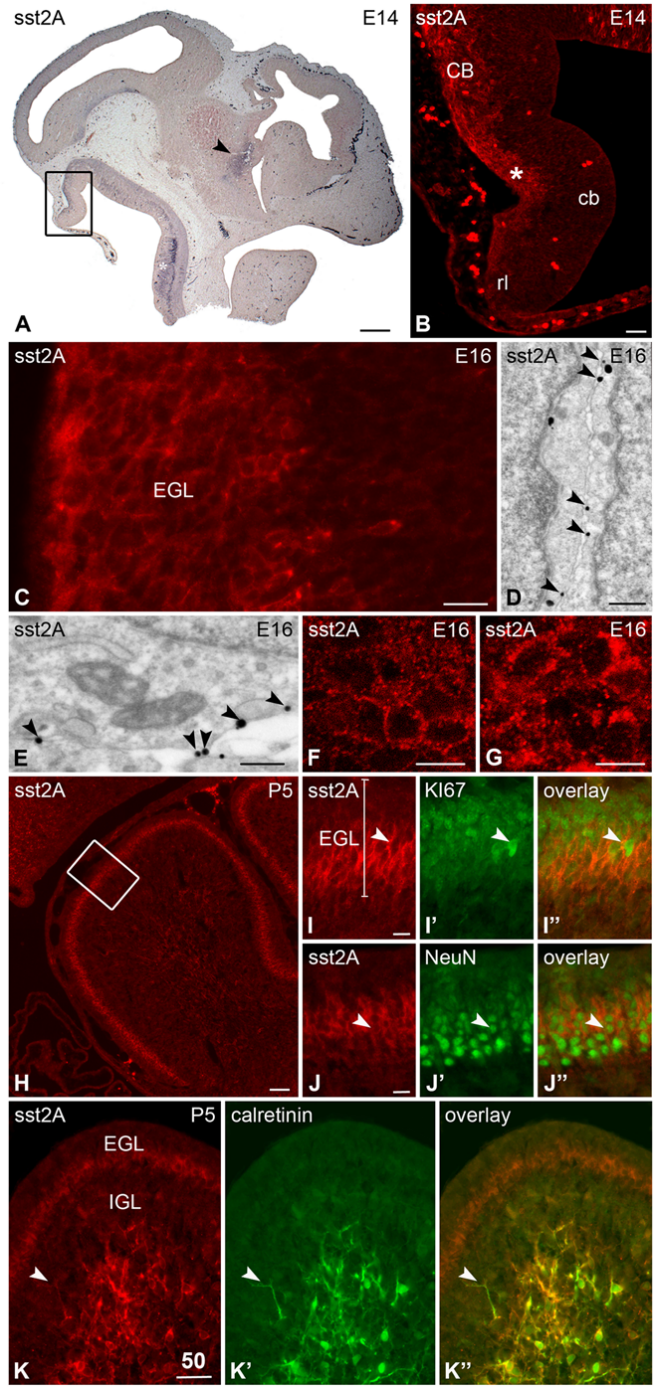
Regional, cellular and subcellular distribution of sst2A receptor immunoreactivity in sagittal sections of the rat cerebellum during pre- and postnatal development. A) At E14, sst2A receptor immunoreactivity is detected in the developing cerebellum (boxed area). Note the strong expression of the receptor in the developing hypothalamus (arrowhead) and rhombencephalon (asterisk). B) The sst2A receptor immunoreactivity is intense at the outer border of the cerebellar neuroepithelium (asterisk) and the adjacent upper component of the rhombic lip (rl). C) At E16, strong cellular sst2A receptor labeling is evident in the dorsal part of the cerebellum, where the progenitors of the external granular layer (EGL) migrate. D,E) Pre-embedding immunogold immunohistochemistry of the sst2A receptor in the developing external germinal layer at E16 illustrates that immunoparticles are predominantly localized at the internal surface of the plasma membrane (arrowheads). F,G) High magnification confocal microscopic analysis of the developing EGL reveals redistribution of surface receptors to intracellular compartments upon agonist stimulation. In control conditions, sst2A receptor immunoreactivity outlines the periphery of neurons (F). Forty minutes after agonist administration, accumulation of immunoreactive puncta in the cytoplasm become evident (G). H) At P5, intense sst2A receptor immunofluorescence is observed in the EGL. I–I″ represent magnification of boxed area in H. The sst2A receptor-immunoreactive neurons (red) are predominantly located in the deep part of EGL (I). The Ki-67-immuonreactive proliferative neurons (green) are distributed predominantly in the superficial EGL (I′). Accordingly only few sst2A receptor-immunoreactive neurons are Ki-67-positive (I″; arrowheads). J–J″) In the EGL, most sst2A receptor-immunolabeled neurons (red in J, J″) are positive for the neuronal-specific nuclear protein NeuN (green in J′, J″) (arrowheads) and demonstrate the post-mitotic nature of sst2A-positive EGL neurons. K, K″) At P5, the large unipolar calretinin-immunoreactive brush cells (green in K′, K″) are sst2A receptor immunoreactive (red in K, K″). Note the colocalization of sst2A receptor and calretinin in a long brush cell process (arrowhead). cb, cerebellar neuroepithelium; CB, cerebellum. Scale bars: A, 250 µm; B, H, K–K″, 50 µm; C, 20 µm, D, 200 nm; E, 400 nm. F, G, I–I″, J–J″, 10 µm.

In the developing cerebellum, immunopositive cells for the receptor located along the outer border of the cerebellar neuroepithelium were apparent as early as E13. At E14, intense receptor immunostaining was detected in the proliferative zone of the upper component of the rhombic lip (Fig. 3). This latter region corresponds to the germinal zone for the progenitors of the external granular layer (EGL). The vast majority of these sst2A positive cells migrating towards the future site of the EGL were post-mitotic, although some of them still expressed the proliferative marker Ki-67. From E14 to P10 the internal sublamina of the EGL strongly expressed sst2A receptor immunoreactivity. At E16, immunogold particles labeling the receptor were visualized by electron microscopy at the inner side of plasma membrane of round or elongated-shaped cells in the EGL (Fig. 3D,E), suggesting that receptors are efficiently targeted to active sites and could be functional. The proportion of membrane-associated receptor was 38.26±2.83% of the total number of receptors. In vivo agonist-induced internalization and consequent redistribution of the receptor in intracytoplasmic domains is a powerful tool to demonstrate that receptors reach plasma membrane and can be activated by agonists, as previously demonstrated in the adult rat brain [30]–[32]. In keeping with results obtained by electron microscopy, incubation of E16 brains with the sst2 receptor agonist octreotide induced redistribution of receptor immunoreactivity in the EGL (Fig. 3F,G). At P5, double-labeling experiments demonstrated that most of the sst2A receptor-expressing cells were not Ki-67-positive (Fig. 3I–I″) but colocalized with the neuronal marker NeuN (Fig. 3J–J″). The sst2A receptor was not expressed within the Purkinje cell layer. Beginning on E18, another important group of sst2A receptor-positive cells appeared to exit rostrally and ventrally from the rhombic lip toward the brainstem. Another pathway appeared to exit the rhombic lip dorsally toward the cerebellar cortex and disperse in a fountain-like spray. Receptor expressing cells were then gradually found in the developing internal granular layer (IGL). At P5, nearly all sst2A receptor-positive cells within the IGL also expressed the calcium-binding protein calretinin (Fig. 3K–K″). Conversely, nearly all of calretinin-positive cells were also positive for the somatostatin receptor. These sst2A receptor-expressing cells are most likely glutamatergic interneurons named unipolar brush cells because of calretinin expression and their oval-shaped and large perikaryon size displaying a single thick dendrite and a single axon. After P10, sst2 receptor immunoreactivity was no more detected in the cerebellum.

**Figure 4:**
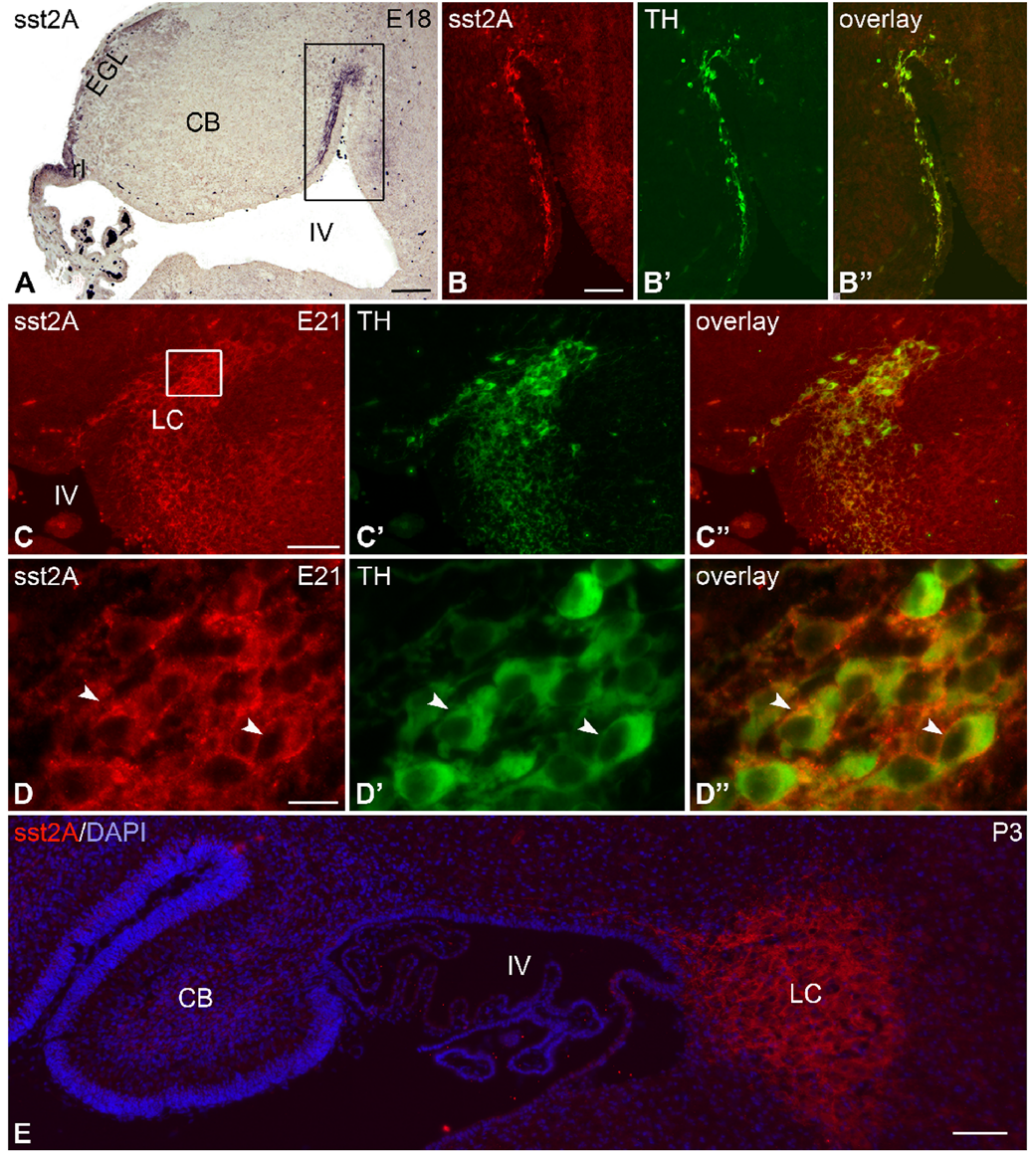
Regional and cellular distribution of sst2A receptor immunoreactivity in the rat developing locus coeruleus. (A) At E18, strong sst2A receptor immunoreactivity is found not only in the rhombic lip (rl) and external granular layer (EGL) but also in the rostro-ventral part of the cerebellum between the cerebellar ventricular area (IV) and the ventral hindbrain (boxed area). B–B″) The large, elongated sst2A receptor-immunoreactive cells (red in B, B″) lie parallel with the ventricular surface. These neurons also express tyrosine hydroxylase (TH; green in B′, B″), a marker of catecholaminergic neurons. C) At E21, intense sst2A receptor immunoreactivity (red in C, C″) is observed in the developing locus coeruleus (LC) and overlap with TH immunolabeling (green in D′,D″). D represents high magnification of boxed area in C. Note that intense sst2A receptor immunoreactivity (red) outlines the periphery of TH-positive (green) neurons (arrowheads). E) At P3, the locus coeruleus exhibits also strong sst2A receptor immunoreactivity (red). The blue labeling represents DAPI staining. CB, cerebellum; IV, fourth ventricle. Scale bars: A, 200 µm; B–B″, C–C″, E, 100 µm; D–D″, 20 µm.

As mentioned previously, a subpopulation of cells strongly immunoreactive for the sst2A receptor, displaying a large and round cell body with usually a single immunoreactive process were visible at E18 between the ventral part of the cerebellum and the ventral part of the hindbrain (Fig. 4A). Interestingly, these cells also expressed tyrosine hydroxylase (TH) (Fig. 4B–B″), suggesting that they could be the *anlage* of the locus coeruleus and/or sublocus coeruleus. In keeping with this observation, strong receptor immunoreactivity displaying a honeycomb cellular pattern was present in the locus from E18. Most of these cells were also immunoreactive for TH (Fig. 4C–E). The peak of receptor expression was observed at E21 in this region and persisted until adulthood.

**Figure 5:**
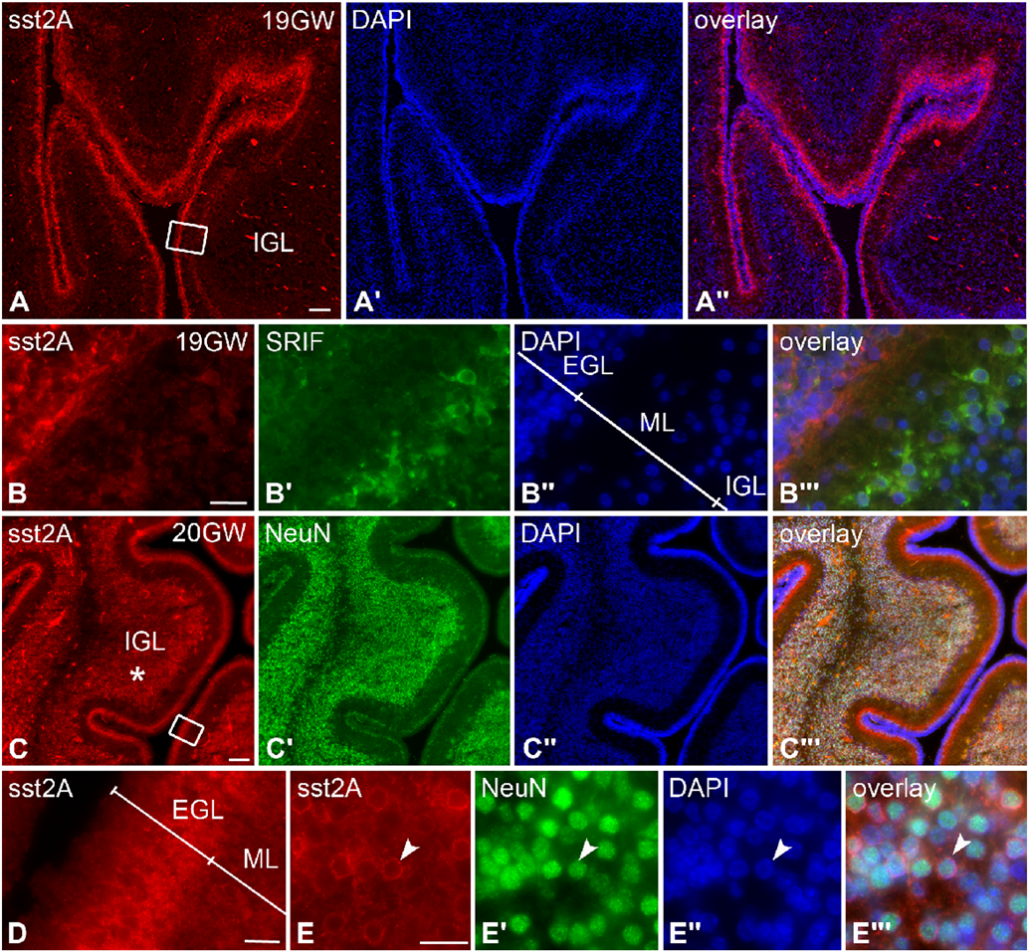
Regional and cellular distribution of sst2A receptor immunoreactivity on coronal sections of the prenatal human cerebellum. A) At GW 19, intense receptor immunoreactivity (red in A, A″) is observed in the deep part of the external granular layer (EGL). Note the large number of DAPI-positive cell nuclei (blue in A′, A″) in the superficial EGL. B–B″′ represent high magnification of boxed area in A. The sst2A receptor immunoreactivity (red in B, B″′) is mainly distributed in the deep part of the EGL whereas SRIF-immunoreactive cells (green in B′, B″′) are mainly located in the deep part of the molecular layer (ML). C–C″′) At GW 20, the high density sst2A receptor immunoreactivity (red in C, C″′) in the deep EGL is still present. In addition, intense receptor immunolabeling is detected in the internal granular layer (IGL; asterisk) and overlap with NeuN-immunoreactive cells (green in C′–C″′). D represents magnification of boxed area in C. The sst2A receptor is expressed in cells bodies located in the deep part of the EGL. E–E″′) In the IGL, the vast majority of NeuN- (green in E′, E″′) and DAPI- (blue in E″, E″′) positive cell nuclei are outlined by sst2A receptor immunoreactivity (red in E, E″′) (arrowheads), suggesting that the receptor is expressed by migrating granule cells. Scale bars: A–A″, C–C″′, 100 µm; B–B″′, D, E–E″′, 20 µm.

The expression of the sst2A receptor was also examined in the developing human cerebellum. Similar to the finding in rodents, the deep part of the EGL was strongly immunoreactive at gestational week (GW) 19 (Fig. 5). At this developing stage, SRIF immunoreactive fibers were also evident in the EGL and SRIF neuronal cell bodies were present in the deep part of the molecular layer (Fig. 5B–B″′). At GW 20, sst2A receptor labeling was still intense in the deep part of the EGL (Fig. 5D). Of interest, a very large proportion of cells (around 80%) expressing the neuronal marker NeuN was sst2A receptor-immunoreactive in the IGL and are likely to correspond to migrating granule cells (Fig. 5C–C″′, E–E″′). This receptor expression pattern persisted until birth but, like in rodents, completely disappeared at adulthood.

### Mesencephalon–Diencephalon

The first cells immunoreactive for the sst2A receptor appeared in the lateral component of the superior colliculus at E13. Receptor expressing cells were then visualized in the inferior colliculus at E14. At E16, a dense network of immunoreactive cells was observed in the entire superior colliculus. These cells expressed the β-tubulin marker and often displayed one or two long processes. In the inferior colliculus a similar pattern of immunoreactive neurons was observed although their density was less important than in the superior part of the nucleus. In these two regions, labeled cells and processes persisted until adulthood.

**Figure 6:**
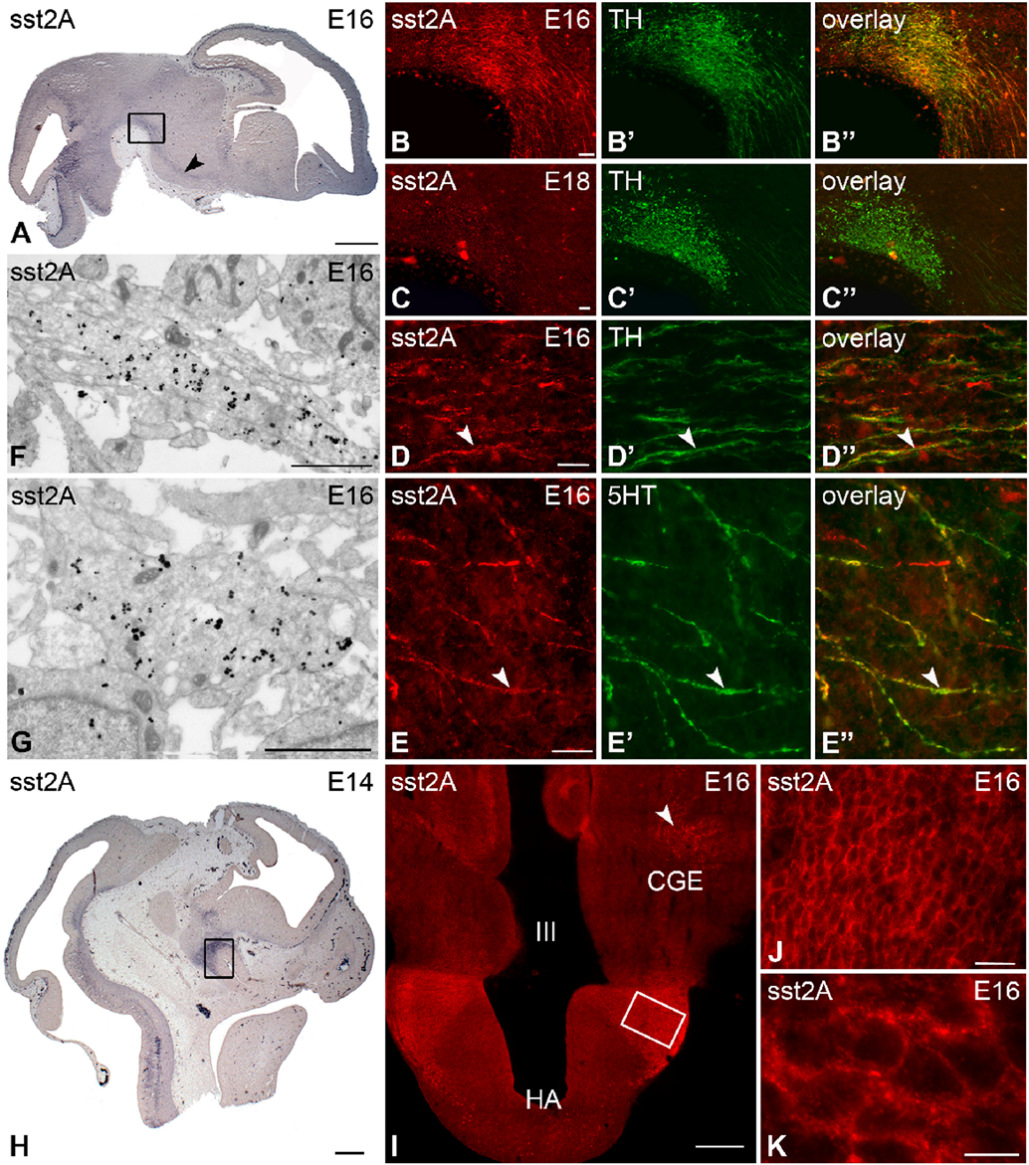
Regional, cellular and subcellular localization of sst2A receptor immunoreactivity on sagittal (A–H) and coronal (I–K) sections of the rat mesencephalon and diencephalon between E14 and E18. A) At E16, intense sst2A receptor immunoreactivity is observed in the substantia nigra (SN; boxed area) and along the medial forebrain bundle (mfb; arrowhead). B–B″) At E16, the sst2A receptor (red in B, B″) and tyrosine hydroxylase (TH) (green in B′, B″) immunoreactivities extensively overlap both in the SN and in emerging processes of the mfb. C–C″) At E18, sst2A receptor immunoreactivity is dramatically decreased in both the SN and the mfb. D–D″) High magnification microscopic images illustrate numerous sst2A receptor-immunoreactive fibers (red in D,D′) in the mfb at E16. Some of them are TH-positive (green in D′,D″) (arrowheads). E–E″) Some sst2A receptor-immunolabeled axons (red in E, E″) of the mfb express 5-HT (green in E′, E″) (arrowheads). F,G) Pre-embedding immunogold immunohistochemistry of the sst2A receptor in the mfb at E16 illustrates very high density of immunoparticles in axons (F) and growth cone-like structures (G). Note that although the majority of immunoparticles are intracellular, some are found associated to the plasma membrane. H) At E14, intense sst2A receptor immunolabeling is observed on sagittal sections in the developing hypothalamus (boxed area). I) Illustration of receptor immunoreactivity on coronal section at the level of hypothalamic area at E16. Note the receptor immunoreactivity in the caudal ganglionic eminence (CGE) (arrow). J,K) J represents magnification of boxed area in I. At high magnification, sst2A receptor immunoreactivity is found at the periphery of numerous hypothalamic neurons. III, third ventricle; HA, hypothalamic area. Scale bars: A, 500 µm; B–B″, C–C″, 50 µm; D–D″, E–E″, J, 20 µm; F, G, 1 µm; H, 250 µm; I, 200 µm; K, 10 µm.

Beginning at E15, intense sst2A receptor immunoreactivity was detected in the ventral tegmental area (VTA) and the substantia nigra (SN) (Fig. 6). A peak of labeling was observed at E16 in these two regions. Receptor immunoreactivity colocalized with TH immunoreactivity in both neuronal cells and processes (Fig. 6B–B″). A bundle of sst2A receptor positive axons running in the dorsal part of the VTA/SN also expressed TH. The receptor expression, however, remarkably stopped at E18 in these different regions (Fig. 6C–C″). Between E14 and E16, fibers running ventrally from the dorsal part of the rhombencephalon to the developing medial forebrain bundle were strongly immunopositive for the receptor. In this latter region, sst2A immunoreactive fibers were positive for TH (Fig. 6D–D″) or for 5-HT (Fig. 6E–E″), and nearly all of TH or 5-HT fibers were immunopositive for the receptor. Clear receptor immunoreactivity was observed along the length of both types of axons and extended to the growing cones. At the ultrastructural level in the medial forebrain bundle, structures resembling growth cones and axons displayed very high densities of intracytoplasmic but also membrane–associated immunogold particles (Fig. 6F,G). After E16, the receptor fluorescent labeling progressively decreased both in term of intensity and of density of immunoreactive fibers. Only a few fibers were still positive for the receptor at E18 in the medial forebrain bundle.

At E14, numerous cells and processes, strongly immunoreactive for the receptor, appeared in the anterior and lateral hypothalamic area (Fig. 6H–K). From E16 onward, the labeling became fainter and more diffusely distributed throughout the neuropil, affecting most of the developing hypothalamic nuclei except for the most caudal part of the arcuate nucleus. This receptor-immunoreactive distribution persisted during post-natal development and in adulthood.

### Telencephalon

**Figure 7:**
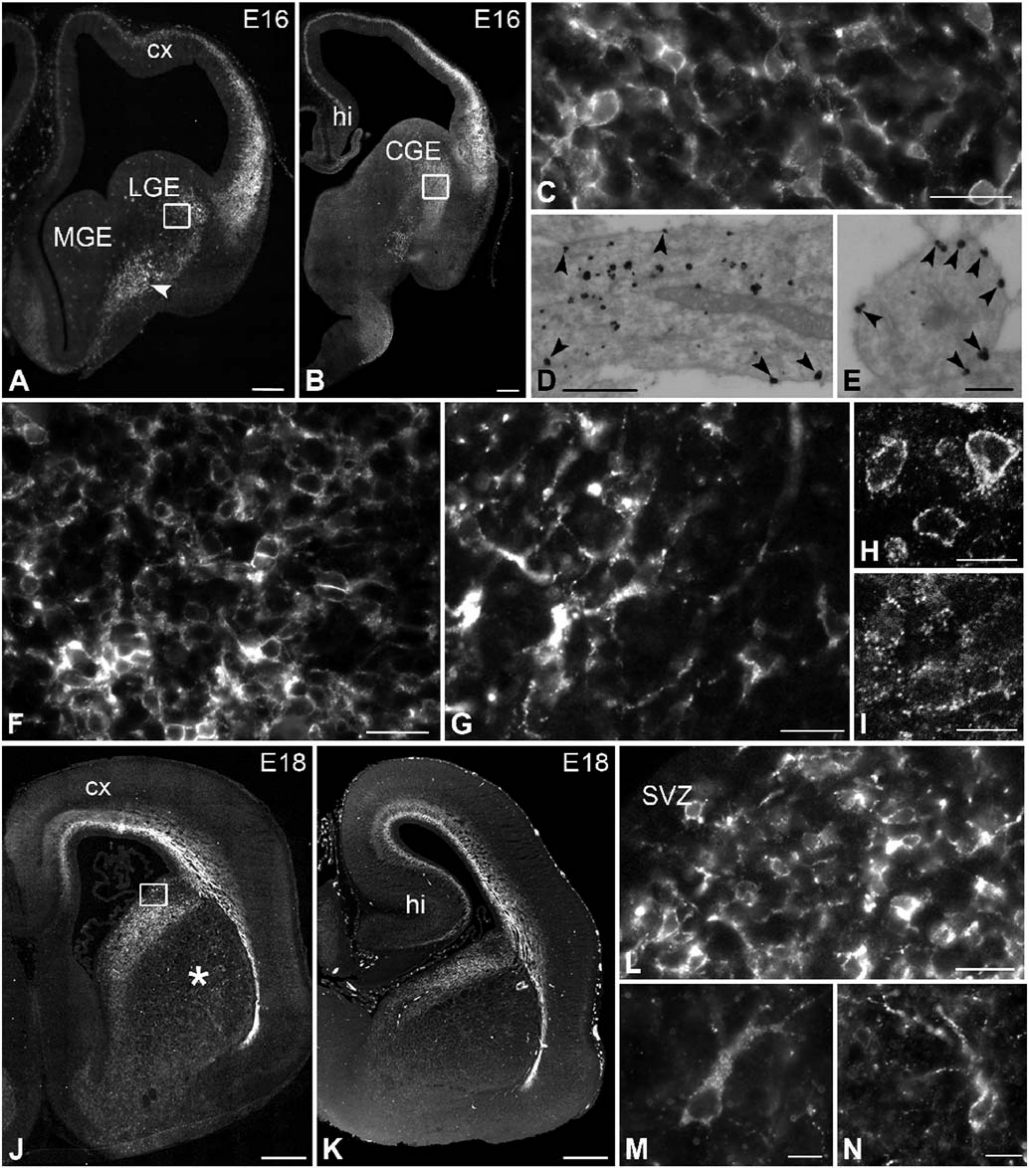
Regional, cellular and subcellular distribution of sst2A receptor immunoreactivity on coronal sections of the rat telencephalon at E16 and E18. A,B) Intense sst2A receptor immunoreactivity is detected at E16 in the post-mitotic areas of the lateral ganglionic eminence (LGE) and the caudal ganglionic eminence (CGE) (B). Note the presence of sst2A receptor immunoreactivity in the cortex (cx) and hippocampus (hi). C represents magnification of boxed area in B and illustrates that the sst2A receptor immunoreactivity is found in cell bodies and short processes in the CGE. D,E) Pre-embedding immunogold immunohistochemistry of the sst2A receptor in the CGE illustrates that high density of immunoparticles are localized intracellularly. However, sst2A receptor-immunoreactive particles are also found in association with the plasma membrane (arrowheads in D). Note that in a neuronal process the majority of the immunoparticles are membrane-associated (arrowheads in E). F represents magnification of the boxed area in A. Numerous cells are immunoreactive for sst2A in the LGE. G represents high magnification of the area labeled with an arrow on A and illustrates that fibers are also sst2A receptor-immunolabeled. H,I) High magnification confocal microscopic analysis in the CGE demonstrates redistribution of receptors upon agonist stimulation. In control conditions, sst2A receptor immunoreactivity outlines the periphery of cells (H). Forty minutes after agonist administration, receptor immunoreactivity is confined to small puncta in the cytoplasm (I). J,K) At E18, intense sst2A receptor immunoreactivity is observed in the dorso-medial part of the caudate-putamen in rostral (J) and caudal (K) sections close to the ventricular surface. Scattered sst2A receptor immunoreactivity is also evident in the medial part of the developing caudate-putamen (asterisk). L represents magnification of boxed area on J. The sst2A receptor immunoreactivity is observed in large number of cells and their short processes in the dorsal caudate-putamen. Note the lack of sst2A receptor immunoreactivity in the subventricular zone (SVZ). M,N are high magnifications from the area labeled with asterisk on J. The sst2A receptor is expressed in neuronal perikarya and processes in the medial part of the caudate-putamen. Scale bars: A, B, 200 µm; C, F, G, L, 20 µm; D, 500 nm; E, 250 nm; H, I, M, N, 10 µm; J, K, 500 µm.

Beginning at E16, numerous post-mitotic neurons were detected in the lateral and caudal ganglionic eminence (Fig. 7). They were small with several short processes, strongly immunoreactive for the receptor and distributed in the dorso-lateral part of the developing caudate-putamen, but sparing the proliferative zone bordering the ventricle. There was a clear demarcation between the distribution of this immunoreactive neuronal population and the one found in the cerebral cortex, i.e. the cortico-striatal junction was devoid of sst2A-immunoreactive neurons. Unlike other developing brain areas, the caudal ganglionic eminence displayed a relatively low proportion (10.28±0.48%) of membrane-associated receptors as detected by electron microscopy (Fig. 7D,E). Redistribution of receptor immunoreactivity was however observed upon agonist stimulation in this region (Fig. 7H,I). In frontal sections, a stream of immunoreactive cells emanating from the ventral part of the caudate-putamen was observed in the ventral parts of the nucleus accumbens and the olfactory tubercle as well as in the ventral part of the lateral septum. In more caudal sections passing through the caudate-putamen this stream was less impressive, although moderate densities of immunoreactive cells were found in ventral regions including the bed nucleus and the hypothalamus. From E16 to P3, a dense population of immunoreactive neurons was distributed in the dorso-medial part of the caudate-putamen (Fig. 7J–N). Here, neuronal cells expressing the receptor were post-mitotic like in other developing structures and were not located in the vicinity of the ventricle. Immunoreactive cells and dendrites as well as axonal fibers were found pervading the globus pallidus, the nucleus accumbens and the olfactory tubercle. Weakly immunoreactive fibers and cells were also visualized in the medial and lateral septum nuclei as well as in the vertical and horizontal limbs of the diagonal band. More caudally, cell and processes expressing the sst2A receptor were found in the amygdaloid nuclei. After P3, the receptor staining became weaker and more diffuse in these regions and displayed the same pattern of distribution as observed at adulthood.

**Figure 8:**
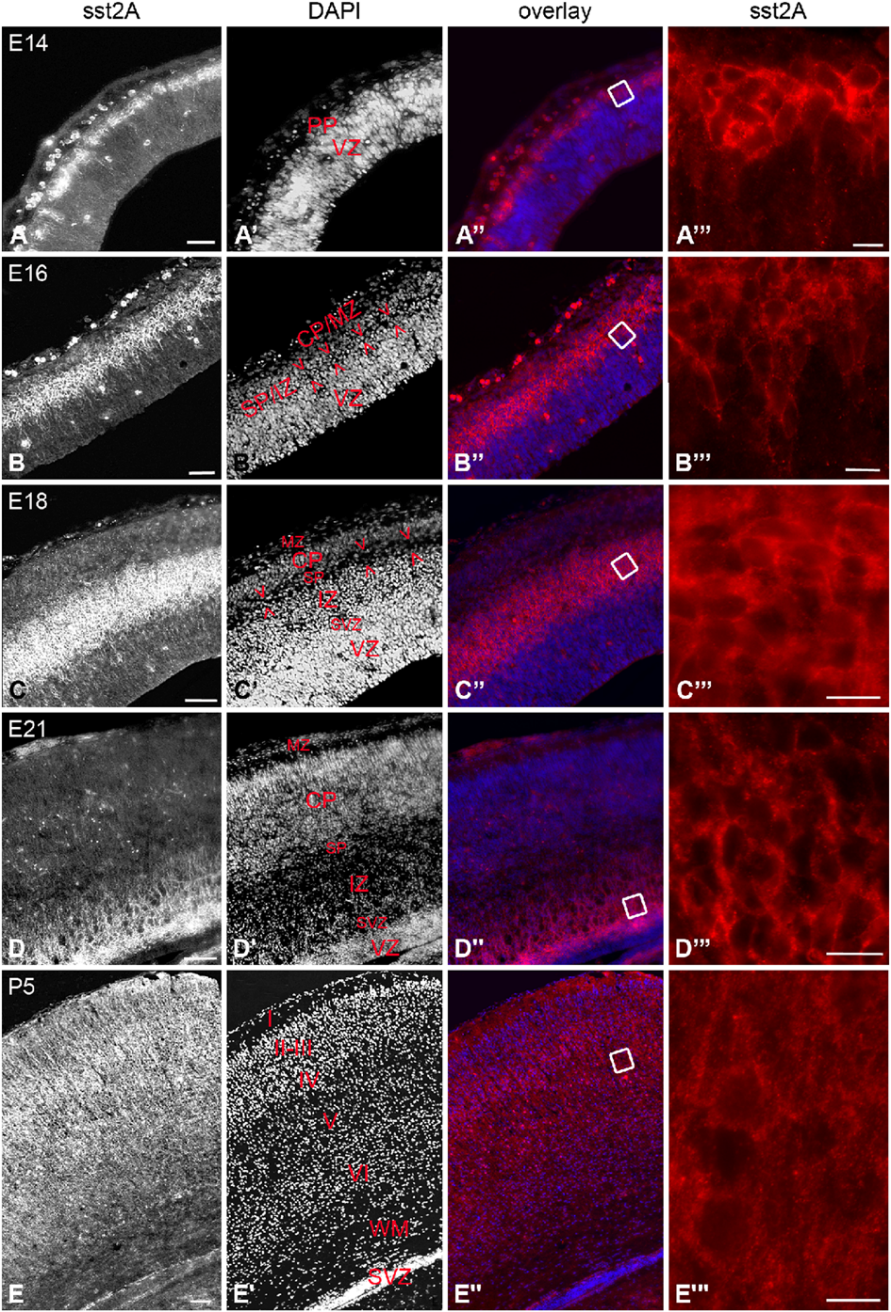
Immunofluorescence of the sst2A receptor in coronal sections through the rat neocortical wall between E14 and P5. A–A″′) At E14, the sst2A receptor immunoreactivity is detected in the preplate (PP). Receptor immunoreactivity is observed in cell bodies and basal processes perpendicular to the pial surface (A″′). B–B″′) At E16, intense receptor immunoreactivity is confined to neuronal cells located in the subplate/intermediate zone (SP/IZ; defined by arrowheads). Immunolabeling is located in cell bodies and small processes of closely packed and presumably migrating neurons (B″′). C–C″′) At E18, the sst2A receptor immunoreactivity is confined to cells in the intermediate zone but absent from the adjacent subplate (defined by arrowheads). D–D″′) At E21, sst2A receptor immunoreactivity is concentrated in the subventricular zone (SVZ) and the adjacent deep part of IZ. Immunoreactivity is apparent in cell bodies and radially oriented processes (D, D″′). E) At P5, the sst2A receptor immunoreactivity is diffusely distributed over the neuropil. The labeling intensity decreases towards the deep layers. At high magnification, receptor immunoreactivity appears diffusely distributed within the neuropil (E″′). A″′, B″′, C″′, D″′ and E″′ represent magnifications of boxed areas on A″, B″, C″, D″ and E″, respectively. CP/MZ, cortical plate/marginal zone; CP, cortical plate; MZ, marginal zone; VZ, ventricular zone; I–VI, cortical layers I to VI; WM, white matter. Scale bars: A–A″, B–B″, 50 µm; C–C″, D–D″, E–E″, 100 µm; A″′–E″′,10 µm.

**Figure 9:**
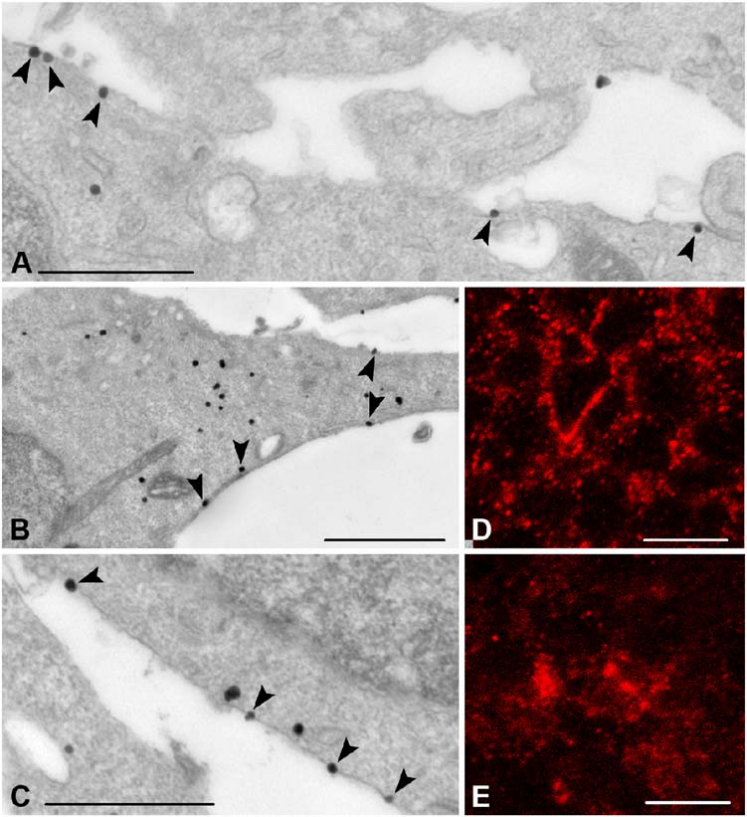
Subcellular localization of sst2A receptor immunoreactivity in neocortical cells at E16. A–C) Pre-embedding immunogold immunohistochemistry of the sst2A receptor in the developing cortex at E16 demonstrates localization of immunoparticles at the internal surface of the plasma membrane (arrowheads). D,E) High magnification confocal microscopic analysis reveals agonist-induced redistribution of surface receptors to intracellular compartments. In control conditions, sst2A receptor immunoreactivity outlines the periphery of cells (D). Forty minutes after agonist administration, accumulation of immunoreactive puncta in the cytoplasm become evident (E). Scale bars, A, C, 500 nm; B, 1 µm; D, E, 10 µm.

The developing cerebral cortex represented the brain structure with the highest receptor expression throughout prenatal development. Sst2A receptor-immunoreactive cells were first detected in the preplate at E13–14 (Fig. 8A–A″′). At E16, sst2A receptor immunoreactivity was restricted to the subplate/intermediate zone in which post-mitotic neurons are migrating (Fig. 8B–B″′). At E18, the immunolabeling was very intense in the intermediate zone (Fig. 8C–C″′), in densely packed neurons as well as short processes. At E21, intense receptor immunoreactivity became apparent in the subventricular zone and the adjacent deep intermediate zone (Fig. 8D–D″′). With increasing fetal ages, more fibers and processes tended to become immunoreactive. Although lightly stained, dispersed bipolar cells with processes oriented perpendicularly to the cortical surface were also apparent above and below the intermediate zone. Only few immunoreactive cells were seen in the upper part of the cortical plate and receptor expression was not visualized in the marginal zone. After birth, the receptor immunoreactivity pattern and expression changed remarkably. The labeling was no more observed in the subventricular zone but became diffuse, homogeneously distributed over the neuropil and formed a gradient across the cortical layers, from intense in the superficial layers to very low in the deepest layers (Fig. 8E–E″′). With cortical maturation, this diffuse labeling progressively shifted to the layers V–VI. Around P14 and thereafter, distribution of receptor immunoreactivity was similar to that reported in adulthood, i.e. enriched in the upper part of the layer V and the deeper part of the layer VI with somatodendritic immunoreactive profiles and ascending dendrites only occasionally apparent, scattered in the diffuse sst2A receptor immunostaining. At E16, the presence of receptor at the plasma membrane was confirmed by immunogold electron microscopy and represents 30.64±3.09% of the total number of receptors (Fig. 9A–C). Agonist treatment at this age triggered internalization of receptor immunoreactivity thereby demonstrating clearly that surface receptors expressed by these neurons can be activated (Fig. 9D,E).

**Figure 10:**
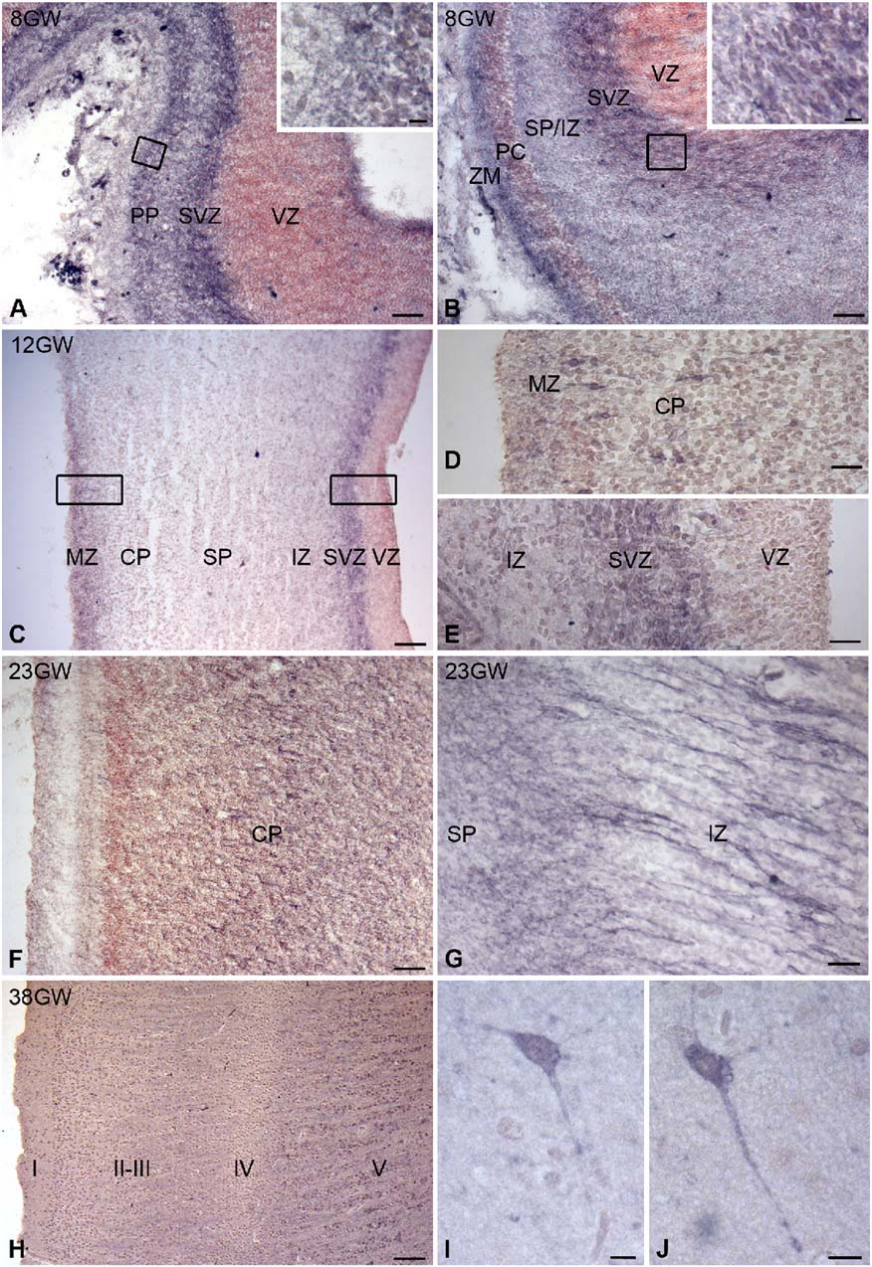
Regional and cellular distribution of sst2A receptor immunoreactivity in the human developing cortex. A,B) Embryonic sagittal sections at GW 8 at the level of the medial cerebral cortex (A) reveals numerous receptor immunoreactive cell bodies (purple color) in the preplate (PP) (inset box) and in the subventricular zone (SVZ). By contrast the ventricular zone (VZ) is devoid of receptor immunoreactivity. The red color is due to the counterstaining of sections with neutral red. In the lateral part of the medial cerebral cortex (B), sst2A receptor immunoreactivity is detected in the marginal zone (MZ), cortical plate (CP), subplate/intermediate zone (SP/IZ) and SVZ (inset box). C–E) At GW 12 in coronal sections, albeit less intense, the pattern of receptor immunoreactivity is comparable to that observed at GW 8 with higher signals in the MZ (C,D) and SVZ (C,E). D and E are magnifications of boxed areas in C at the level of the MZ and the SVZ, respectively. Note in D that some bipolar neurons expressing the sst2A receptor are visible in the CP and in E that patches of labeling are observed in the SVZ contiguous to the VZ. F, G) In coronal sections at GW 23, the labeling is present in neurons of CP (F) as well as in presumably post-mitotic migrating neurons in the IZ (G). H–J) In coronal sections at birth, the labeling is diffusely distributed in layers II–III and V. In this latter layer some neurons positive for the sst2A receptor are also observed (I,J). Scale bars: A, B, D, E, G, 25 µm; C, F, H, 100 µm; Inset in A,B and I,J, 10 µm.

Because sst2A receptor immunoreactivity was striking in the developing cortical wall in rats, we next investigated whether the sst2A receptor is also expressed in the developing human cerebral cortex (Fig. 10). Receptor immunoreactivity was first detected at GW 8 in the cortical wall. Because maturation of the human cerebral cortex follows a latero-medial gradient with an offset of approximately 15 days, the distribution of receptor immunoreactivity was different between the lateral and the medial parts of the cerebral cortex. In the latter, the bulk of receptor expression was visualized in the preplate and in the subventricular zone contiguous to the ventricular zone [33]. The labeling was clearly absent in the ventricular zone. In the lateral cortical *anlage*, where the first cortical plate neurons have migrated, the labeling was mostly detected in the subventricular zone, at the limit with the germinal zone. A lighter immunoreactive band was also observed superficially in the marginal zone. Scattered immunoreactive cells were evident in the subplate and cortical plate. From GW 12 onward, the cerebral wall is composed of the germinative zone (divided into a ventricular and a subventricular zone), the intermediate zone (future white matter), the subplate, the cortical plate, and the superficial marginal zone (future layer I). At GW 12, sst2A receptor immunoreactivity was observed in several layers in the frontal lobe. In both medial and lateral cortices, a dense immunoreactive band was observed in the subventricular zone. Scattered, but strongly immunoreactive neurons, often displaying a process perpendicularly oriented to the cortical surface, were observed in the marginal zone as well as in the outer limit of the cortical plate. This labeling was more intense in the lateral than in the medial parts of the developing cortex. In the intermediate zone, neuronal cells tangentially orientated to the cortical surface displayed faint sst2A receptor labeling.

**Figure 11:**
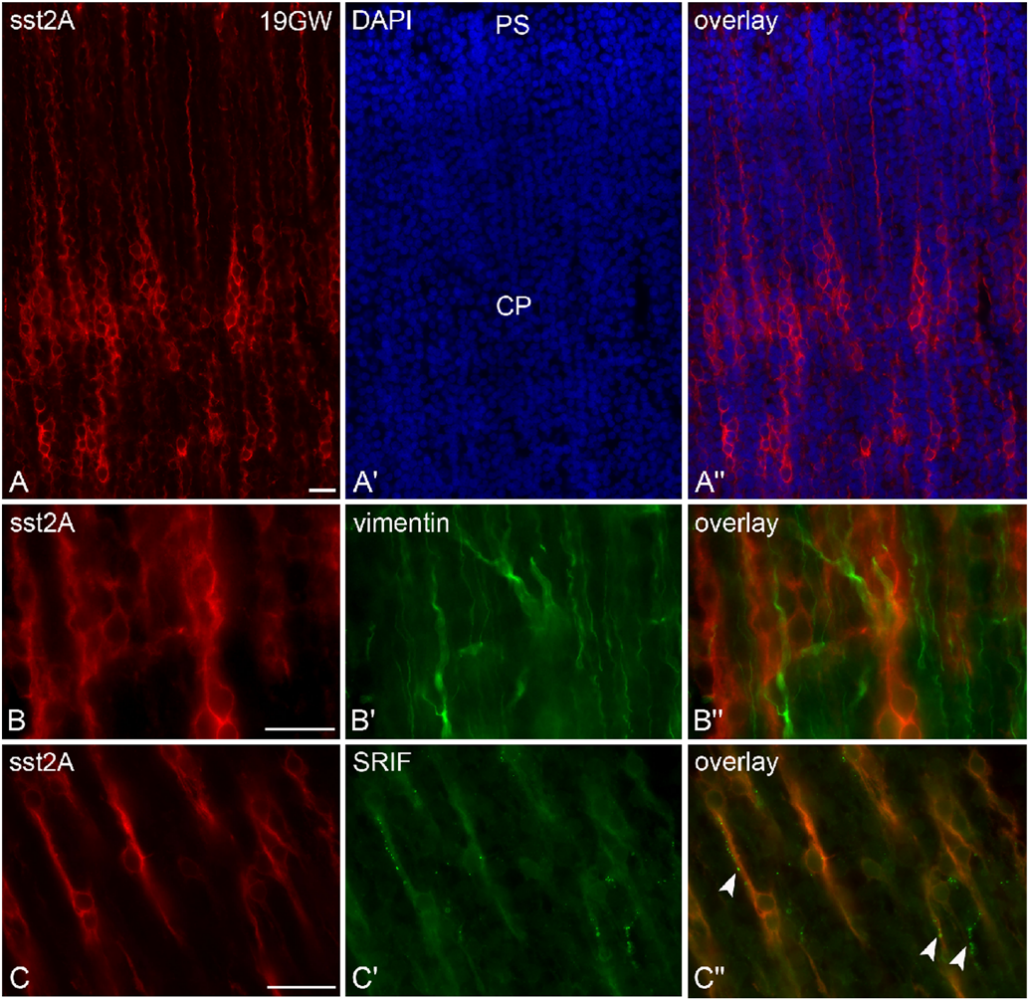
Regional and cellular distribution of the sst2A receptor immunofluorescence in the human cerebral cortex at GW 19. A–A″) Intensely labeled sst2A receptor-immunoreactive neurons (red in A′–A″) form chain-like clusters in the middle part of the cortical plate (CP). Note that long sst2A receptor-immunoreactive radial processes reach the pial surface (PS). B–B″) Receptor-immunolabeled cells and processes (red in B, B″) are closely apposed by vimentin-positive processes (green in B′B″), suggesting migration of sst2A-labeled cells on radial glia. C–C″) Sst2A receptor-immunoreactive processes (red in C, C″) are contacted by fibers that are immunoreactive for SRIF (green in D, D″) (arrowheads), the endogen ligand of the receptor. Scale bars: 20 µm.

At GW 19 in parieto-temporal cortical areas, a slight receptor labeling was still detected in the subventricular zone. More conspicuous were the strongly immunoreactive cells and long processes organized in clusters and chains within the middle part of the cortical plate, and oriented perpendicularly to the cortical surface (Fig. 11A–A″). Numerous processes extended to the marginal zone. Double-labeling experiments clearly demonstrated that these cells and processes were in close apposition with vimentin-positive processes (Fig. 11B–B″), a marker of radial glia, suggesting that these cells represent migrating clusters. At this developmental stage, SRIF-immunoreactive fibers were found running along sst2A receptor positive cell bodies and processes (Fig. 11C–C″).

From GW 21 to GW 23, the receptor labeling detected in the subventricular zone at earlier stages could no longer be detected. A dense labeling of radially oriented neurons was visualized in the layer V. Their apical dendrites reached the layer I and were associated with a diffuse immunolabeling there. In the upper subplate, immunopositive neurons were numerous, whereas they were sparse and dispersed in the inner subplate. The sst2A receptor immunoreactivity within the axonal component of the intermediate zone almost disappeared. A similar distribution of receptor labeling was detected in the visual cortical areas at the same developmental ages. From GW 30 to birth in both fronto-parietal and occipital cortical areas, the receptor labeling became weaker and more homogeneously and diffusely distributed over the layers V–VI, a distribution similar to that observed in the adult human brain [6], [34].

**Figure 12:**
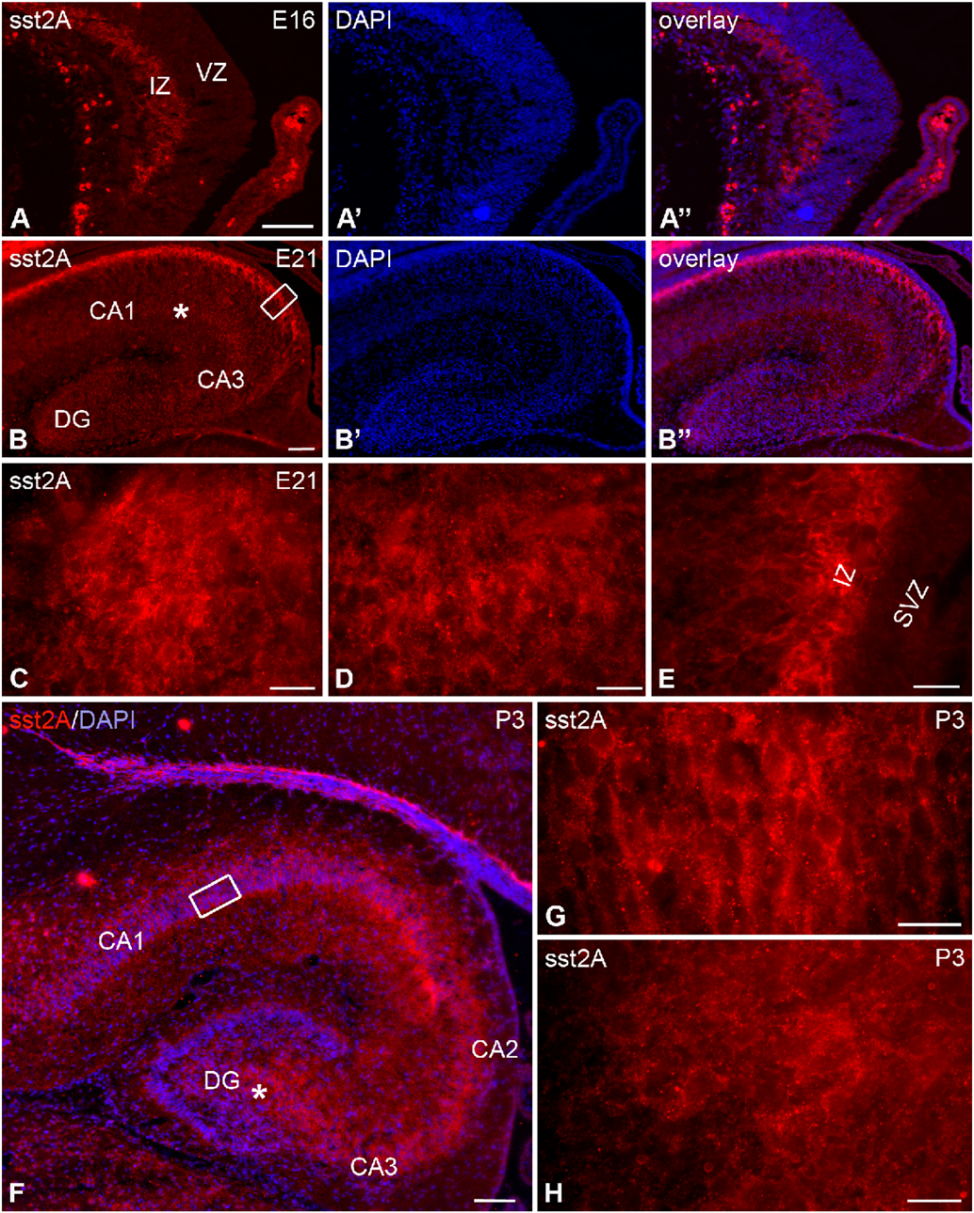
Distribution of the sst2A receptor immunoreactivity in the rat hippocampus during pre- and postnatal development. A–A″) At E16, sst2A receptor immunoreactivity (red in A, A″) is localized in the intermediate zone (IZ) of the hippocampus. Note the lack of immunoreactivity in the ventricular zone (VZ). B–B″) At E21, the most intense immunolabeling is found in the intermediate zone of CA1. In addition, less intense immunolabeling is apparent in the pyramidal cell layer as well as in the strata oriens and radiatum of CA1, in the CA3 and in the developing dentate gyrus (DG). C) In the hilus of the DG, sst2A receptor immunoreactivity appears diffusely distributed. D represents magnification of the area labeled with asterisk in B and illustrates the diffuse sst2A receptor immunolabeling observed in the CA1 pyramidal cell layer. E represents magnification of boxed area in B. The sst2A receptor immunolabeling is intense in cells of the IZ whereas the subventricular zone (SVZ) is devoid of labeling. F) At P3, intense immunofluorescence is detected in the pyramidal layer, strata oriens and radiatum of CA1-3, as well as in the hilus of DG. The molecular layer of dentate gyrus is weakly immunoreactive. G represents magnification of boxed area in F and illustrates the intense sst2A receptor immunolabeling localized in CA1 pyramidal cell bodies and proximal dendrites. H represents magnification of area labeled with asterisk in F and illustrates the diffuse sst2A receptor immunolabeling observed in the hilus of the DG. Scale bars: A–A″, B–B″, F, 100 µm; C, D, E, G, H, 20 µm.

In the rat hippocampal formation, the first immunoreactive cells appeared at E16. They were round with short processes and localized contiguous to the neuroepithelium in the intermediate zone (Fig. 12A–A″). At all embryonic stages from E14 to birth, sst2A receptor immunoreactivity was absent in proliferating precursor cells located in the ammonic neuroepithelium, the primary dentate neuroepithelium or the secondary dentate matrix. Between E16 and E18, the labeling remained strong in the intermediate zone. In addition, weak and diffuse sst2A receptor labeling was apparent in the pyramidal cell layer and in the developing hilus of the dentate gyrus. By E21, there was a substantial increase of immunoreactive labeling in the pyramidal cell population as well as in the stratum oriens and radiatum, while the intermediate zone still displayed the highest amount of receptor immunoreactivity (Fig. 12B–B″, C–E). At that fetal age, granule cells of the dentate gyrus began to express the sst2A receptor. At P3, sst2A receptor labeling was widely distributed in all strata and subfields of the Ammon's horn and the dentate gyrus, albeit more concentrated on the cell bodies and proximal dendrites (Fig. 12F–H). Starting at P10, sst2A receptor immunoreactivity was increasingly visible over more distal portions of pyramidal and granular cell dendrites. By P21, sst2A receptor immunoreactivity was essentially similar to that observed in adulthood, i.e. diffusely distributed over the dendritic fields of principal neurons.

**Figure 13:**
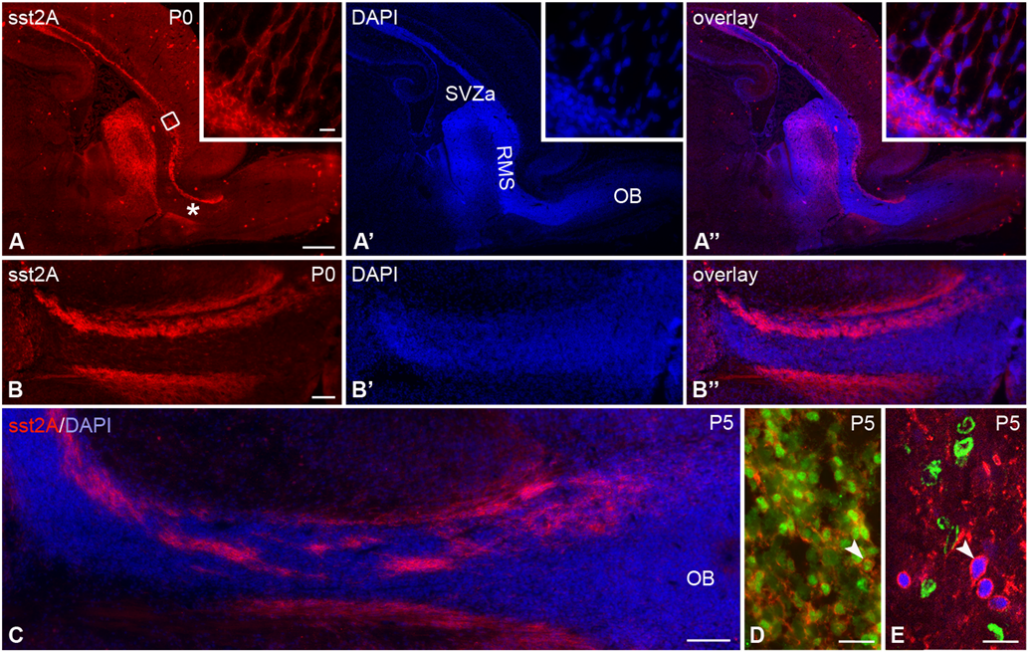
Immunofluorescence of sst2A receptor in the rat perinatal rostral migratory stream. A–A″) In sagittal sections at P0, an intense band of sst2A receptor immunoreactivity is observed from the anterior subventricular zone (SVZa), through the rostral migratory stream (RMS) and ending in the olfactory bulb (OB). From the SVZa, shown in detail in the high magnification insets, chains of immunoreactive neurons perpendicular to the SVZa long axis extend into the white matter of the overlying cerebral cortex. B–B″ represents high magnification of the area labeled with asterisk in A. The sst2A receptor immunoreactive cells are principally localized along the ventral and dorsal surface of RMS. C) High magnification of sst2A receptor immunoreactivity at the entrance of RMS into the olfactory bulb at P5 illustrates immunoreactive cells at the surface of the stream as well as embedded in central position. D) In the dorsal part of the RMS, sst2A receptor-immunoreactive cells (red) contain NeuN labeling in their nuclei (green; arrowhead). E) Sst2A receptor- (red) and NeuN- (blue) double-labeled cells (arrowhead) of the RMS do not contain BrdU immunoreactivity (green), demonstrating that receptor expression is restricted to post-mitotic neurons. Scale bars: A–A″, 500 µm; B–B″, C, 100 µm; D, 20 µm; E, 10 µm.

A very dense but transient population of sst2A receptor-immunoreactive cells was observed between P0 and P10 in the anterior subventricular zone (SVZa) and the rostral migratory stream (RMS) (Fig. 13). These cells were mainly localized at the dorsal part of the SVZa/RMS and therefore represent a subpopulation of this structure. They were positive for the neuronal marker NeuN (Fig. 13D). Furthermore, following a short pulse of BrdU (3 h), the BrdU-positive cells were found negative for sst2A receptor immunoreactivity (Fig. 13E). These results suggest that receptor expression is restricted to migrating neuroblasts. Also, emanating from the border between the SVZa/RMS and the frontal cortex, chains of neurons (from ∼5 to ∼10 cells) were visualized entering the developing white matter towards the deep layers of the cerebral cortex (Fig. 13A–A″ insets). Double-labeling experiments with vimentin, a marker of radial glial cells, clearly demonstrated that sst2A receptor-immunoreactive processes were found in close apposition to glial processes. Before reaching the olfactory bulb, two streams of immunopositive cells were visible in the ventral and dorsal parts of the RMS. However, in the olfactory bulb proper, cells immunoreactive for the receptor were not observed, suggesting that cells migrating radially lose their receptor expression.

### Functional approaches

**Figure 14:**
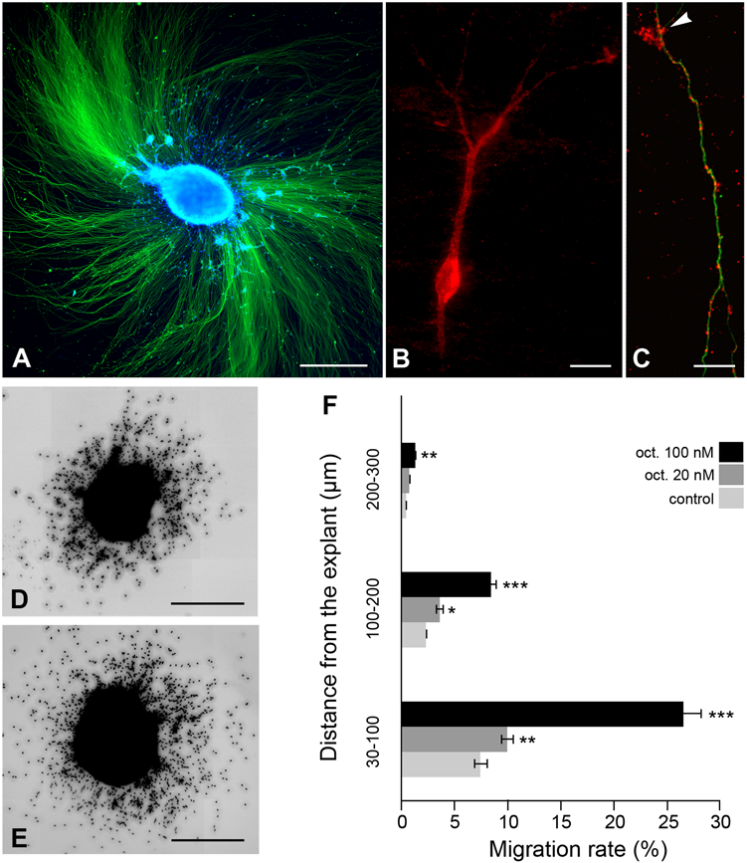
Effect of sst2A receptor activation on *in vitro* granule cell migration. A) Representative image of an external granular layer (EGL) microexplant after 3 days *in vitro* in culture. The core of the explant and surrounding scattered migrating granules cells are labeled with DAPI (blue). Neuronal processes are labeled by neuronal class III β-tubulin immunoreactivity (green). B) In individual granule cells, sst2A receptor immunoreactivity is visible in both neuronal perikarya and processes (red). C) Illustration of sst2A receptor immunolabeling (red) in a β-tubulin-immunoreactive (green) axon. Note the sst2A-immunoreactive puncta in a growth cone structure (arrow). D, E) In comparison to control (D) the number of migrating granule cells is significantly increased in 100 nm octreotide-treated EGL (E) microexplants. The octreotide-induced granule cell migration increase is dose-dependent as revealed by quantitative analysis (F). *p<0.05; **p<0.01; ***p<0.001. Values represent mean±SEM. Scale bars: A, D, E, 500 µm; B, C, 10 µm.

Because the regional and cellular localization of the sst2A receptor suggests a potential role on neuronal migration and differentiation events, we next investigated the effects of receptor activation in well characterized *in vitro* models. To assess the role of the sst2A receptor on cell migration, EGL microexplant cultures were treated with the sst2A receptor agonist octreotide (Fig. 14A). In these cultures, granule cells migrate out without glial support and follow the sequence of their *in vivo* differentiation pattern [35]. Migrating granule cells in EGL explants were sst2A receptor-immunoreactive. The sst2A receptor was localized in cell bodies, cellular processes and growth cones (Fig. 14B,C). In explants treated with octreotide, the number of migrating granule cells and the maximal migration distance from the explant increased considerably when compared to control conditions (Fig. 14D,E). Quantitative analysis indeed revealed a dose-dependent significant increase of the migration rate of EGL neurons in octreotide-treated explants as compared with controls (Fig. 14F). In contrast, neurite outgrowth was similar in octreotide-treated and control EGL microexplants (data not shown).

**Figure 15:**
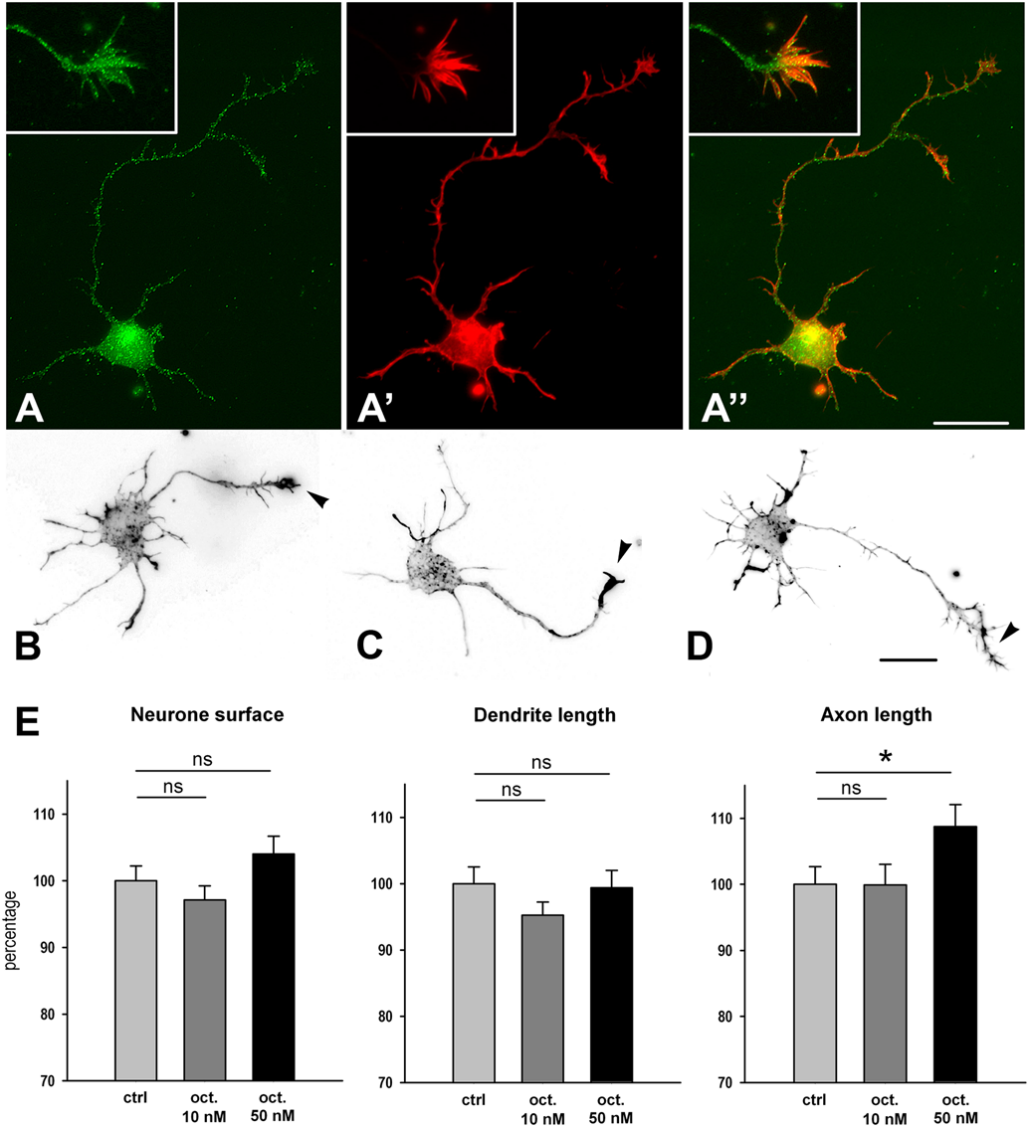
Effect of sst2A receptor agonist on axonal and dendritic patterning. A–A″) Representative image of sst2A receptor localization in a primary hippocampal cell after 24 h *in vitro* in culture. Receptor immunoreactivity (green in A, A″) is present in the cell body and processes. Cell morphology is revealed by actin-binding protein phalloidin (red in A′, A″). Note that sst2A receptor immunoreactivity is also present in growth cones (insets in A–A″). B–D) Representative images of neurons from control (ctrl; B), 10 nM octreotide-treated (10 nM oct.; C) and 50 nM octreotide-treated (50 nM oct.; D) cultures. Arrows depict the axonal process which appears longer when cells are treated with 50 nM octreotide. E) Quantitative analysis reveals that the axon length (right panel) is significantly increased in the 50 nM oct. group when compared to the control group. The mean cell body surface (left panel) and the mean dendritic length (middle panel) are not modified by sst2A receptor agonist treatments. Values (mean±SEM) are expressed in relation to an arbitrary unit (100%) of the control values. *p<0.05; ns, not significant. Scale bars: A–A″, B–D, 20 µm.

Next we analyzed the effect of sst2A receptor activation in the regulation of axonal and dendritic patterning in low-density primary hippocampal neuronal cultures. Young developing neurons displayed intense sst2A receptor-immunoreactivity in cell bodies, dendritic and axonal processes and growth cones (Fig. 15A–A″). Agonist treatment at low concentration (10 nM) did not modify neuronal morphology (Fig. 15B,C). By contrast, quantitative analysis demonstrated a significant increase in the length of axons in the 50 nM octreotide-treated group as compared with controls (+9%; p<0.05) (Fig. 15D, E). The other parameters studied (i.e. cell body surface, length of dendritic processes, number and length of dendritic or axonal branches) were not modified by octreotide treatment.

## Discussion

This study provides the first systematic description of the temporal and spatial expression pattern of a major SRIF receptor, the sst2A subtype, in the pre- and postnatal rat brain and in the developing human cerebral cortex and cerebellum. In addition, we provide evidence that receptor activation has functional consequences on developing neurons. Taken together, our results led to six key findings. First, receptor expression in the rat brain is detected early during ontogenesis (E12) and is restricted to post-mitotic neuronal populations leaving the ventricular zone. Second, the brain structures known to express the sst2A receptor at adulthood also express this receptor during development. Only the medial forebrain bundle, rostral migratory stream and cerebellum expressed the receptor during development but not in adulthood. Third, in contrast to the adult brain in which receptor localization is mainly somatodendritic, some axons and growth cones were found to strongly express the receptor during ontogenesis. Fourth, the localization of receptor at the plasma membrane revealed by electron microscopy and the change of its cellular distribution following agonist challenge argue for functionality of the sst2A receptor during neuronal development. Fifth, activation of sst2A receptors indeed resulted in modification of neuronal migration and neurite patterning in *in vitro* model systems. Sixth, the sst2A receptor was detected both in rat and human developing neuronal structures with remarkable similarities but also differences.

An intriguing feature of the ontogeny of sst2A receptor expression in the brain is its preferential localization in neuronal populations lying in the outer part of the germinal zone at primitive developmental stages in rat and human brain tissues. Double-labeling experiments confirmed that the vast majority of these cells are not proliferative ones and represent young neuroblasts *en route* to populate different regions of the developing brain. The temporal coincidence of sst2 receptors and migrating neurons suggest a role for this receptor during the neuronal migration process. According to this hypothesis, we have demonstrated that activation of the sst2A receptor in EGL microexplants resulted in a dose-dependent increase of granule cell migration. These results are in line with a previous *in vitro* study [19] demonstrating that SRIF-28 increases the migration rate of granule cells near their birthplace, but decreases it near their final destination in the IGL. In both rats and humans, our neuroanatomical data demonstrate the presence of the sst2A receptor in the deep part of the EGL. Together, these studies suggest that the sst2A receptor may be responsible for accelerating the movement of granule cells in the early phase of migration. The termination of migration in the IML could be regulated by another somatostatin receptor in rat, possibly the sst1 receptor [21]. In the developing human cerebellum, it remains to be determined whether the sst2A receptor can assume both roles since in addition to the EGL, it is also observed in the granule cells of the IGL.

The persistent expression of the sst2A receptor in different brain regions following the major migratory events suggests that it could also have a role in neuronal differentiation. In line with this hypothesis, sst2A receptor stimulation induced a moderate but significant increase of the axonal length of hippocampal neurons in culture. Such an effect was not found in EGL microexplants in which sst2A receptor activation increased granule cell migration. Thus depending on the regions and/or developmental time of expression, the sst2A receptor is likely to assume different roles during neuronal maturation.

In addition to the cerebellum, our morphological data suggest involvement of sst2A receptor in neuronal migration in several other brain areas. In the developing rat and human cerebral cortex, the immunoreactive cells were found first in the preplate and later in the deep intermediate/subventricular zone. These particular regions contain the earliest-generated neurons of the cortex that will migrate using the processes of radial glial cells to reach their final position. In both rat and human cortex, sst2A receptor-positive neurons were indeed found in close apposition with vimentin-positive processes. Together, these results suggest that the sst2A receptor could play a role in the early events of the radial migration of cortical excitatory neurons.

In the developing forebrain, numerous sst2A receptor-positive cells were visible in the LGE and CGE. These two areas contribute to the development of numerous brain structures, including the neocortex, striatum, thalamus, septum and olfactory bulb for the former and nucleus accumbens, bed nucleus of the stria terminalis, hippocampus, amygdala, striatum and globus pallidus for the latter [36], [37]. Interestingly, sst2A receptor-expressing cells are indeed present during the course of maturation in most of these brain regions, as illustrated in the striatum at E16, in which strongly immunoreactive cells and processes are visible. Neurons emanating from the LGE and the CGE usually use nonradial migration to reach their final destination suggesting a potential role of the sst2A receptor in the so-called tangential (or neurophilic) neuronal migration [37]. By contrast, the absence of sst2A receptor-expressing cells in the MGE (the major source of cortical interneurons) together with the lack of a sst2A receptor-positive cell stream between the developing subpallium and cerebral cortex (an important pathway of migrating interneurons) do not argue for a major role of this receptor in the migration of GABAergic neocortical interneurons. In line with this observation, using a gene expression microarray analysis on cortical interneuron precursors, the sstr2 gene was found not to be enriched in this population [38]. Of note, cortical interneurons, including the somatostatinergic ones, do not express this receptor at adulthood [39].

Between P0 and P10, a subpopulation of cells in the SVZ and RMS were also sst2A receptor-positive. Cells born in the SVZ migrate through chain migration along the RMS to the olfactory bulb, where they differentiate into local interneurons [40]. Our results clearly established that, like in other developing brain areas, sst2A receptor-expressing cells were post-mitotic neuroblasts. Additional investigations are required to determine whether the sst2A receptor is a marker of a particular subpopulation of migrating neuroblast and to analyze its potential motogenic role in this cell population. It will be also of particular interest to determine the nature and the final destination of sst2A receptor-labeled neuroblasts that seem to detach from the dorsal part of the SVZ and migrate to the white matter and/or the cerebral cortex.

Interestingly, transient expression of the sst2A receptor was observed during the development of specific neuronal populations. Thus, both 5-HT and TH expressing cells were strongly immunopositive for the sst2A receptor between E15 and E17 in the developing raphe nuclei and the VTA/SN, respectively. In contrast, in adult rat brains, these regions were devoid of receptor immunoreactivity. A peak of receptor immunoreactivity was also observed at E16 in TH and 5-HT growing axons in the medial forebrain bundle. The sst2A receptors are distributed on the entire neuronal structure throughout soma-dendrites, axons and all the way to the growth tip. Together these observations suggest that the sst2A receptor may play a role, during a restricted time-window, in the migration and normal positioning of TH and 5-HT neurons as well as in axonal growth and guidance of these two cell populations.

At the ultrastructural level, we found that a significant proportion of sst2A receptors was associated with the plasma membrane. Interestingly, the proportion of membrane-associated receptors in the developing cortex was similar to that in the adult cortex [39]. In our internalization assay experiments, sst2 receptor activation induced redistribution of receptors in different brain areas, indicating that receptors at plasma membrane can be activated by agonists, as previously demonstrated in the adult rat brain [30]–[32]. Taken together, these data clearly suggest that the sst2A receptors are well poised to transduce the effects of endogenous ligands early during embryonic life. The existence of SRIF neurons and significant concentrations of SRIF has been reported in the brain as early as E12 in rat [41]–[46]. In addition, the mRNA encoding cortistatin has also been detected in the developing brain [47]. Endogenous cortistatin may be an additional physiological ligand of the sst2A receptor during brain development. In the human brain, the present study demonstrates the concomitant presence of the receptor and its ligand. SRIF could also be transported from the maternal blood across the placental barrier to the fetus, since somatostatin immunoreactivity has been detected in human amniotic fluid [48].

The sst2A receptor distribution in rat and human developing cortex and cerebellum exhibited similarities but also differences. In this latter structure, the deep layer of EGL intensely expressed the sst2A receptor in both species. In the IGL, however, migrating granule cells in humans were receptor-immunopositive. By contrast, in rats IGL, unipolar brush cells but not granule cells expressed the receptor. Unipolar brush cells are a unique type of glutamatergic interneurons that play an important role in vestibulo-cerebellar circuitry [49]. They are produced in the rhombic lip, a region with high density of sst2A receptor-positive neuroblasts, and migrate to their final destination during late embryonic and early postnatal development [50].

In the cerebral cortex, the sst2A receptor was located in the preplate at early developmental stages in both rats and humans. As soon as the cortical plate was formed, the sst2A labeling extended to the entire intermediate zone/subplate in the rats whereas it was enriched in the outer part of the germinal zone in humans. The latter location in humans emphasizes the possible role of sst2A receptor on very early post-mitotic neurons arising all along the neuronal proliferation period which last about 3 months. Later in development the sst2A cortical labeling evolved towards the deep layers as detected in the adult in both species.

In conclusion, our observations strongly suggest that in addition to its neuromodulatory role in the adult brain, the sst2A receptor participates in the development and maturation of specific neuronal populations during brain ontogenesis in both rats and humans.

## Materials and Methods

### Animals

Pregnant Sprague Dawley rats were purchased from Janvier Laboratories (Le Genest St Isle, France). The breeding was made during the night and the day after insemination was considered as embryonic day 0.5. The day of birth was designated as P0. Three to four male and female per stage were studied at the following ages: E10, E12, E13, E14, E16, E18, E21, P0, P3, P5, P7, P10 and P21. All experiments were carried out in accordance to the ethical principles of the Institut National de la Santé et de la Recherche Médicale (INSERM).

### Human samples

Brain tissues of human embryos and fetuses of 8, 9, 12, 19, 21, 23, 30, 35 and 38 gestational weeks (GW) were used in this study [51], [52]. Written consent was routinely obtained from parents, and approval for the study were given by the French National Ethics Committee (CCNESVS, approval number 90 294) in accordance to French laws and international regulations (Declaration of Helsinki, 2000). Cerebral tissues were fixed in 4% paraformaldehyde in phosphate buffer (pH 7.4, 0.12 M) for 8 h, rinsed and cryoprotected in phosphate buffer supplemented with 20% sucrose. Serial sagittal or coronal 12 µm-thick cryostat sections were obtained and processed for immunocytochemistry as mentioned below.

### Immunohistochemical experiments

#### Primary antibodies

The sst2A receptor was immunolocalized by using an antiserum raised in rabbit against the C-terminal segment 330–369 of the human protein (1/5000) that specifically recognized sst2A antigens in rat and human brain sections by immunohistochemistry as previously demonstrated [6], [53]–[55]. Immunocytochemical controls for sst2A receptor labeling consisted of adsorption of the antibody with 50 mg/mL of sst2A receptor-GST fusion proteins overnight at 4°C, and incubation with the preimmune in place of the immune serum.

Several antibodies were used in double-labeling experiments to characterize cells and processes expressing the sst2A receptor. For a comprehensive list of the antibodies and their characteristics, see Table 1. Controls for double-labeling staining included omission of the primary antibodies to test for nonspecific binding of the secondary antibodies and incubation with one primary but both secondary antibodies to demonstrate the absence of cross-labeling.

**Table 1:** List of Primary Antibodies.

Name	Immunogen	Source	Catalog Number	Host and type	Clone/Code	Dilution	Species reactivity	Ref.
Calretinin	human recombinant calretinin	Swant, Bellinzona, Switzerland		goat polyclonal	CG1	1∶1000	human, rat	[62]
GAD67	recombinant GAD67	Chemicon, Temecula, CA	MAB5406	purified mouse monoclonal IgG2a	1G10.2	1∶1000	human, rat	[63], [64]
GFAP	purified GFAP from pig spinal cord	Sigma-Aldrich, St. Louis, MO	G6171	purified mouse monoclonal	G-A-5	1∶1000	human, rat	[65], [66]
Ki-67	recombinant human Ki-67 peptide	BD Biosciences Pharmingen, San Diego, CA	556003	purified mouse monoclonal IgG1κ	B56	1∶200	human, rat	[67], [68]
NeuN	purified cell nuclei from mouse brain	Chemicon	MAB377	purified mouse monoclonal IgG1	A60	1∶1000	human, rat	[69]
Neuronal Class III β-Tubulin	rat brain microtubules	Covance, Berkeley, CA	MMS-435P	purified mouse monoclonal IgG2a	TUJ1	1∶1000	human, rat	[70], [71]
Serotonin	serotonin conjugated to BSA	Chemicon	MAB352	rat monoclonal	YC5/45	1∶500	human, rat	[72]
Somatostatin	human SRIF C-terminus	Santa Cruz Biotechnology, Santa Cruz, CA	sc-7819	purified goat polyclonal IgG	D-20	1∶500	human, rat	[6], [55]
Somatostatin 2A receptor	recombinant human sst2A receptor	Lone Helboe		rabbit polyclonal		1∶5000	human, rat	[6], [39]
TH	TH purified from PC12 cells	Chemicon	MAB318	ascites mouse monoclonal IgG1κ	LNC1	1∶1000	human, rat	[73], [74]
Vimentin	purified vimentin from pig eye lens	Santa Cruz Biotechnology	sc-6260	mouse monoclonal IgG1	V9	1∶400	human, rat	[75], [76]

#### Tissue preparation

Pregnant females were sacrificed by cervical dislocation and the abdominal cavity was opened to remove embryos. The brains of embryos were quickly dissected and fixed in 4% paraformaldehyde (PFA) in 0.12 M phosphate buffer, pH 7.4 (PB) overnight at 4°C. Pups were anesthetized with isofluorane and intracardially perfused for 5 min with 4% PFA in PB. Brains were immediately removed and postfixed in the same fixative overnight at 4°C. All specimens were then cryoprotected for 2 days in a 10% sucrose solution in PB at 4°C. Brains were immerged in a solution of 7.5% gelatin, 10% sucrose in PB for 1 h at 37°C. They were after embedded in a block of the same solution for 1 h at 4°C. Brains were frozen in liquid isopentane at −70°C and stored at −80°C until sectioning. Parasagittal or coronal sections (10 µm of thickness) were cut on a cryostat and collected on Superfrost plus slides (Microm Microtech, Francheville, France).

#### Immunoperoxidase procedure

Cryostat sections were air-dried and underwent 10-minute rinses in 0.1 M phosphate buffer saline, pH 7.4 (PBS). They were then washed twice 10 min in 0.2% gelatin/0.25% Triton X-100 in PBS. Primary and secondary antibodies were incubated in 0.25% Triton X-100 with 10% goat normal serum in PBS to block the nonspecific binding sites and aid permeabilization. Primary sst2A antibody (1∶5000) was incubated overnight at 4°C. After rinsing in PBS, the rabbit anti-sst2A antiserum was detected using a 90 min incubation in a biotinylated goat anti-rabbit IgG (1∶200, Sigma-Aldrich, St. Louis, MO) solution followed by a 90 min incubation in an avidin-biotin-peroxidase complex reagent (1∶400, Amersham Pharmacia Biotech, Buckinghamshire, UK) at room temperature. Peroxidase enzyme activity was revealed using 3, 3′- diaminobenzidine tetrahydrochloride (DAB; 0.01%) in 0.05 M Tris buffer saline, pH 7.6, in the presence of 0.002% H_2_O_2_ and 0.6% nickel ammonium sulfate. Finally, the sections were rinsed in distilled water and dehydrated through graded ethanols, treated with xylene and coverslipped with Permount (Fisher Scientific, Pittsburgh, PA) for light microscopic examination.

#### Double labeling immunofluorescence procedure

Mixtures of primary antibodies were incubated overnight at 4°C in a 10% donkey normal serum/0.2% gelatin/0.25% Triton X-100 in PBS solution. The following day, sections were rinsed three times in 0.2% gelatin/0.25% Triton X-100 in PBS, followed by a 90 min incubation in a mixture of appropriate secondary antibodies. Secondary antibodies used were cyanine 3 (Cy3)-conjugated donkey anti-rabbit (1∶300, Jackson ImmunoResearch, West Grove, PA), Alexa Fluor 488 (A488)-conjugated donkey anti-mouse, A488-conjugated donkey anti-goat and A488-conjugated donkey anti-rat (1∶200, Invitrogen, Carlsbad, CA). They were incubated in 10% donkey normal serum/0.2% gelatin/0.25% Triton X-100 in PBS. After three further rinses in PBS, sections were stained for few seconds with DAPI (1∶1000), rinsed in PBS and coverslipped with Fluoromount-G (SouthernBiotech, Birmingham, AL) for fluorescence microscopic examination.

### Sst2A receptor internalization essay

The brains of three E16 embryos were quickly dissected and immediately immersed for 40 minutes at 37°C, 5% CO_2_ in a solution of artificial cerebrospinal fluid (25 mM KCl, 2 mM KH_2_PO_4_, 25 mM Hepes, 37 mM D-glucose, 10 mM MgSO_4_ and 175 mM sucrose) containing 100 µM sst2A receptor agonist octreotide (SMS 201–995) or PBS (control). After 40 minutes, brains were fixed in 4% PFA in PB overnight at 4°C and processed for sst2A receptor immunofluorescence staining as describe above. Brain sections were analyzed using a Zeiss Axio Observer inverted microscope equipped with a LSM 5 Exciter confocal scanning system (Carl Zeiss, Jena, Germany).

### BrdU incorporation assay

Three P5 rat pups were injected intraperitoneally with a solution of 5-bromo-2′-deoxyuridine (BrdU, Sigma; 50 mg/kg body weight) 3 h prior to perfusion with 4% paraformaldehyde. After fixation, brains were processed for immunohistochemistry as described above. Cryostat sections were air dried, denatured 30 min in 2 N HCl in PBS, and rinsed three times in PBS. BrdU incorporation was visualized by immunofluorescence using a rat anti-BrdU monoclonal antibody (1∶200, Abcam, Cambridge, UK) and an A488-conjugated donkey anti-rat antibody (1∶200, Invitrogen).

### Electron microscopy

Immunocytochemical procedures for the detection of the sst2A receptor at the ultrastructural level were performed as previously described [30], [39]. Briefly, the brains of embryos were quickly dissected and fixed in 4% PFA with 0.05% glutaraldehyde in PB for 2 h at 4°C. Brains were post-fixed overnight in 4% PFA in PB at 4°C, washed in 0.01 M phosphate-buffered saline, pH 7.4 (PBS) and embedded in 4% Agarose type LM SIEVE (Euromedex, Souffelweyersheim, France). Sagittal sections were cut on a vibratome at 50 µm and collected in PBS. Sections were equilibrated in 25% sucrose and 10% glycerol in 0.05 M PB, frozen rapidly in isopentane, cooled in liquid nitrogen and thawed in PBS at room temperature. Sections were then incubated in 5% normal goat serum (NGS) in PBS for 30 min and incubated for 16 h at room temperature in anti-sst2 receptor antibody diluted 1∶800 in PBS containing 1% NGS. After washing in PBS, they were incubated for 2 h in a 1∶100 dilution of NANOGOLD- goat anti-rabbit IgG (1.4 nm in diameter; Nanoprobes, Stony Brook, NY) in PBS containing 2% of bovine serum albumin-c and 0.2% of cold water fish gelatin. Sections were then washed in PBS and post-fixed in 1% glutaraldehyde in PBS for 10 min. After repeated washing in PBS and 0.1 M sodium acetate buffer, pH 7.0, gold labeling was intensified using a silver enhancement kit (HQ Silver; Nanoprobes) for 5 min in the dark at room temperature. Sections were finally washed in acetate buffer and then in PB. Immunogold-treated sections were post-fixed in 1% osmium tetroxide in PB 0.1 M for 10 min at room temperature. After washing three times in PB, they were dehydrated in an ascending series of ethanol, which included 1% uranyl acetate in 70% ethanol. They were then treated with propylene oxide twice for 10 min, equilibrated overnight in Durcupan ACM (Fluka, Buchs, Switzerland), mounted on glass slides and cured at 60°C for 48 h. Areas of interest were cut out from the slide and glued to blank cylinders of resin. Immunoreactive samples identified on thick sections were cut in semithin sections (1 µm) and then in ultrathin sections on a Reichert Ultracut S microtome. Ultrathin sections were collected on pioloform-coated single-slot grids. Sections were stained with lead citrate and examined with a Philips CM120 electron microscope. The subcellular distribution of sst2 receptor in the developing cortex, cerebellum and ganglionic eminence was analyzed as previously described [30], [39]. Plasma membrane-associated and intracellular immunoparticles were counted. The relative distribution of the membrane-associated and intracellular receptors was calculated in relation to the total number of receptors per cells and expressed in percentage.

### *In vitro* experiments

#### Microexplants culture

External granular layer microexplants cultures of P3 rats were prepared as described previously [35], [56]. To test the effect of sst2A receptor activation on granule cell migration, octreotide (0, 20, 100 nM) was added to the culture medium once per day on day *in vitro* (DIV) 1–3. Explants were fixed on DIV 3 with 4% PFA and 4% sucrose in PB. For permeabilization and blocking of unspecific binding of antibodies, the explants were pre-incubated with 10% normal donkey serum and 0.2% gelatin in PBS for 30 min. Cultures were then incubated in rabbit sst2A receptor (1∶5000) and mouse neuronal class III β-tubulin (1∶500, Covance, Berkeley, CA) primary antibodies overnight at room temperature, then in Cy3-conjugated donkey anti-rabbit (1∶300, Jackson ImmunoResearch) and A488-conjugated donkey anti-mouse (1∶200, Invitrogen) secondary antibodies in 1% NDS/0.2% gelatin/0.1% saponin in PBS for 90 min. Explants were stained for few seconds with DAPI (1∶1000), rinsed in PBS and coverslipped with Fluoromount-G (SouthernBiotech) for fluorescence microscopic examination.

Migration analysis was performed using the MetaMorph software (Molecular Devices, Downingtown, PA) as described previously [56], [57]. Twenty three control, twenty-four 20 nM octreotide-treated and nineteen 100 nM octreotide-treated explants were analyzed. To measure migration rates, concentric areas at increasing distances from the explant border were delimited. The number of DAPI-labeled pixels within each area was counted and then expressed as a percentage of the total number of pixels. To evaluate the overall rate of neuronal migration, the total number of DAPI-labeled pixels surrounding the explants was counted. Neuritic length was estimated by laying out a circle containing approximately 90% of the ß-III-tubulin positive neurites. Values from control and octreotide-treated explants were analyzed by Kruskal-Wallis nonparametric test followed by Dunn's multiple comparison test using GraphPad Prism version 4.03 (GraphPad Software, San Diego, CA). The value of p<0.05 was considered as statistically significant.

#### Low density cultures of primary hippocampal neurons

Primary hippocampal neuron culture was prepared as described previously [58]–[60]. Two hours after plating, octreotide (0, 10 or 50 nM) was added to the neurons in Petri dishes and incubated for 24 h in the presence of feeder glia. Neurons were fixed with 4% PFA and 4% sucrose in PB for 20 minutes before permeabilization and blocking with 0.066% saponin, 0.22% gelatin in PB. Cells were incubated for 1 h in rabbit sst2A receptor (1∶5000) and mouse neuronal class III β-tubulin (1∶500, Covance) primary antibodies in 1% NDS/0.2% gelatin/0.1% saponin in PBS, followed by 45 min incubation in A488-conjugated donkey anti-rabbit (1∶200, Invitrogen) and Cy5-conjugated donkey anti-mouse (1∶500, Jackson ImmunoResearch) secondary antibodies together with phalloidin-A546 (1∶250, Invitrogen). Coverslips were mounted using Fluoromount-G (SouthernBiotech) containing Hoechst 33342 (10 µg/mL, Sigma).

Morphological analysis was performed as described previously [61]. Images of phalloidin-A546-labeled neurons were acquired with a Zeiss Axio Observer microscope (Carl Zeiss). Two hundred and seventy nine control neurons, 276 10 nM octreotide-treated and 231 50 nM octreotide-treated neurons were analyzed. Number and length of processes, including axonal and dendritic branches, as well as areas of cell bodies were measured using Image J (National Institutes of Health, Bethesda, MD). Among the primary processes, the axon was defined as the longest process, whereas the other processes were classified as dendrites. Values from control and octreotide-treated explants were analyzed by Kruskal-Wallis nonparametric test followed by Dunn's multiple comparison test using GraphPad Prism version 4.03. The value of p<0.05 was considered as statistically significant.
